# Monitoring of cancer ferroptosis with [^18^F]hGTS13, a system xc- specific radiotracer

**DOI:** 10.7150/thno.101882

**Published:** 2025-01-01

**Authors:** Abraham Moses, Rim Malek, Mustafa Tansel Kendirli, Pierre Cheung, Madeleine Landry, Marco Herrera-Barrera, Abbas Khojasteh, Monica Granucci, Syed. A. Bukhari, Jody E. Hooper, Melanie Hayden-Gephart, Scott J. Dixon, Lawrence D. Recht, Corinne Beinat

**Affiliations:** 1 Department of Radiology, Molecular Imaging Program at Stanford (MIPS), Stanford University School of Medicine, Stanford, CA, 94305, USA.; 2 Department of Neurology and Neurological Sciences, Stanford University School of Medicine, Stanford, CA, 94305, USA.; 3 Stanford Institute for Stem Cell Biology and Regenerative Medicine, Stanford University School of Medicine, Stanford, CA, 94305, USA.; 4 Department of Neurosurgery, Stanford University School of Medicine, Stanford, CA, 94305, USA.; 5 Department of Pathology, Stanford University School of Medicine, Stanford, CA, 94305, USA.; 6 Department of Biology, Stanford University, Stanford, CA, 94305, USA.

**Keywords:** system xc-, ferroptosis, PET imaging, [^18^F]hGTS13

## Abstract

Glioblastoma multiforme (GBM) is the most common and aggressive primary brain tumor in adults, characterized by resistance to conventional therapies and poor survival. Ferroptosis, a form of regulated cell death driven by lipid peroxidation, has recently emerged as a promising therapeutic target for GBM treatment. However, there are currently no non-invasive imaging techniques to monitor the engagement of pro-ferroptotic compounds with their respective targets, or to monitor the efficacy of ferroptosis-based therapies. System xc-, an important player in cellular redox homeostasis, plays a critical role in ferroptosis by mediating the exchange of cystine for glutamate, thus regulating the availability of cysteine, a crucial precursor for glutathione synthesis, and influencing the cellular antioxidant defense system. We have recently reported the development and validation of [^18^F]hGTS13, a radiopharmaceutical specific for system xc-.

**Methods:** In the current work, we characterized the sensitivity of various cell lines to pro-ferroptotic compounds and evaluated the ability of [^18^F]hGTS13 to distinguish between sensitive and resistant cell lines and monitor changes in response to ferroptosis-inducing investigational compounds. We then associated changes in [^18^F]hGTS13 uptake with cellular glutathione content. Furthermore, we evaluated [^18^F]hGTS13 uptake in a rat model of glioma, both before and after treatment with imidazole ketone erastin (IKE), a pro-ferroptotic inhibitor of system xc- activity.

**Results:** Treatment with erastin2, a system xc- inhibitor, significantly decreased [^18^F]hGTS13 uptake and cellular glutathione content *in vitro*. Dynamic PET/CT imaging of C6 glioma-bearing rats with [^18^F]hGTS13 revealed high and sustained uptake within the intracranial glioma and this uptake was decreased upon pre-treatment with IKE.

**Conclusion:** In summary, [^18^F]hGTS13 represents a promising tool to distinguish cell types that demonstrate sensitivity or resistance to ferroptosis-inducing therapies that target system xc-, and monitor the engagement of these drugs.

## Introduction

Ferroptosis is a form of regulated cell death that is characterized by the iron-dependent accumulation of membrane lipid peroxides, resulting in oxidative damage and plasma membrane rupture 1,2. Ferroptosis has gathered interest in the cancer research community as it is a unique mechanism of cell death that is mechanistically and morphologically distinct from other forms of cell death (e.g., apoptosis and classic necrosis). Targeting ferroptosis holds great promise owing to the potential to activate this mechanism selectively in cancer cells 3,4. This approach holds particular potential in the treatment of glioblastoma (GBM), the most common primary brain tumor in adults that still is associated with a poor overall survival 5. The current standard of care for patients with GBM includes surgery followed by a combination of radiation and temozolomide chemotherapy 6,7. Unfortunately, these aggressive therapeutic strategies have achieved only limited success, underscoring the need to investigative alternative therapeutic approaches. A recent report showed that certain GBM genotypes are associated with lipidome remodeling that renders them more susceptible to lipid peroxidation, effectively priming these cells for ferroptosis, both *in vitro* and *in vivo* 8.

**Figure 1:**
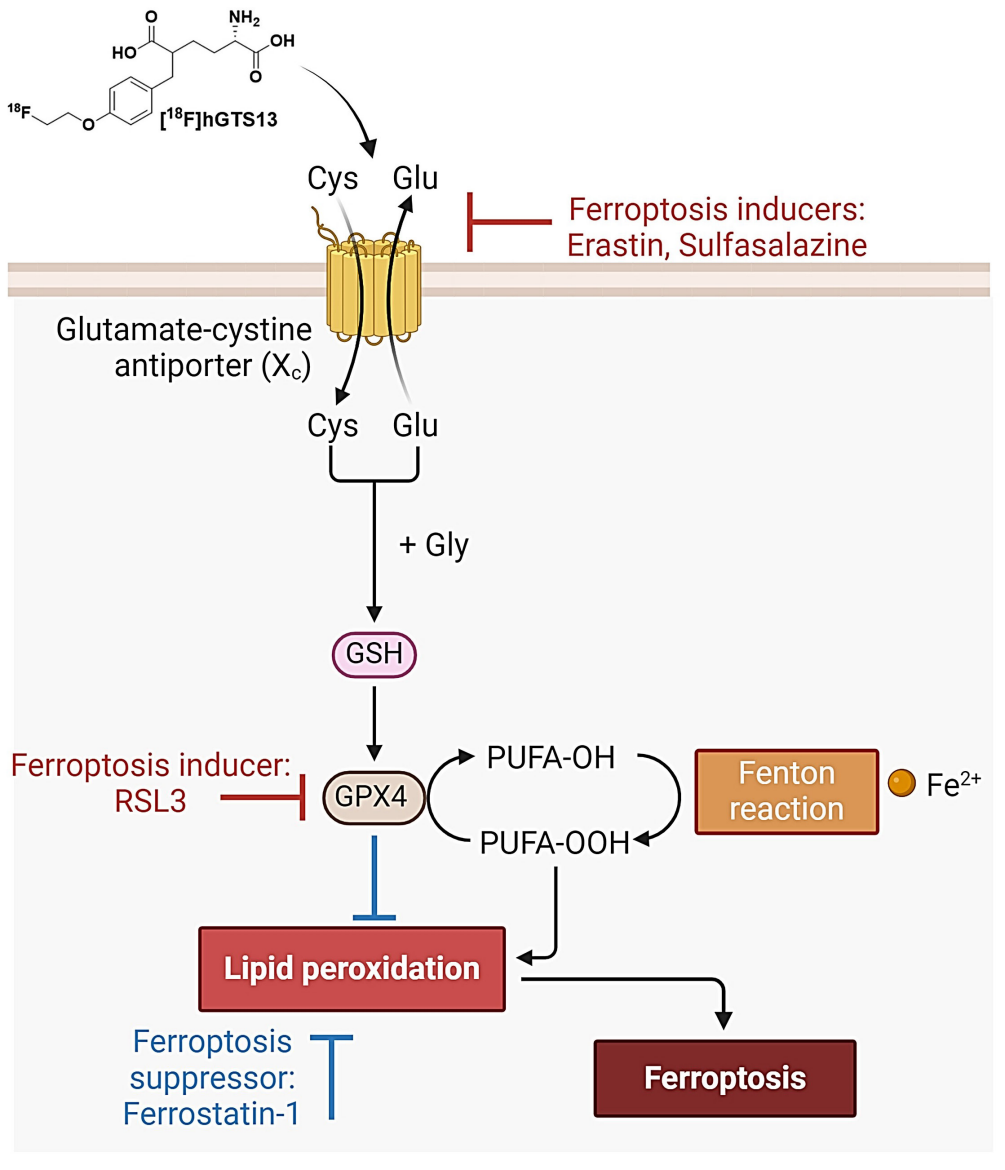
** Overview of ferroptosis.** System xc imports cystine which enables the biosynthesis of glutathione (GSH). GSH is then used by glutathione peroxidase 4 (GPX4) to prevent the accumulation of lethal lipid ROS by reducing reactive polyunsaturated fatty acids (PUFA) phospholipid hydroperoxides (PUFA-PL-OOH) to non-reactive and non-lethal PUFA phospholipid alcohols (PUFA-PL-OH). PUFA-PLs are oxidized by labile Fe(II) and Fe(II)-dependent enzymes. Ferroptosis may be induced *via* pharmacological inhibition of system xc (with erastin/sulfasalazine) or inhibition of GPX4 (with RSL3). Ferroptosis can be suppressed by treatment with ferrostatin-1. [^18^F]hGTS13 is a radiotracer that is specifically transported *via* system xc-.

System xc- is a plasma membrane cystine-glutamate antiporter that imports extracellular cystine in exchange for intracellular glutamate 9. Cystine is a crucial precursor for glutathione biosynthesis, thus impacting the cellular antioxidant defense system and ultimately influencing susceptibility to ferroptosis 10. In this context, the small molecule erastin inhibits system xc-, resulting in cysteine starvation, glutathione (GSH) depletion, and ferroptotic cell death 1,11,12. Indeed, it was the identification of erastin that resulted in the pioneering discovery and definition of ferroptosis 1. Another pharmacological strategy to induce ferroptotic cell death is inhibition of glutathione peroxidase 4 (GPX4) with RSL3 12. While erastin directly inhibits system xc- and induces cystine starvation, RSL3 inhibits the GSH-consuming enzyme GPX4, which normally converts lipid peroxides to lipid alcohols (Figure 1).

Despite the promise of ferroptosis, and the large amount of research centered on inducing ferroptosis in glioma and other cancers, there are no non-invasive imaging technologies that can be used to assess which patients would benefit from ferroptosis-inducing therapy, monitor drug engagement, and predict outcomes. Thus, molecular imaging of system xc- has the potential to 1) identify and select patients with appropriate transporter activity that would be suitable candidates for ferroptosis induction; 2) assess drug engagement and inhibition of system xc- in living subjects; and 3) monitor the efficacy of targeting this emerging mechanism of cell death in cancer. We have recently reported the development and validation of [^18^F]hGTS13, a homo-glutamate radiotracer that is specific for system xc- (Figure 1) and demonstrated acceptable biodistribution in pre-clinical PET imaging studies 13. Importantly, [^18^F]hGTS13 demonstrates several advantages over existing system xc- radiotracers, such as [^18^F]FSPG, including: 1) incorporation of a UV-active group, thus greatly facilitating radiosynthesis and quality control; 2) improved transporter specificity; and 3) reduced uptake in multiple immune cell types and activation states 13. In this study, we report the initial testing of [^18^F]hGTS13 in the context of cancer ferroptosis and further validate its uptake in orthotopic syngeneic rat models of glioma, a cancer that shows promise for ferroptosis inducing therapy.

## Materials and Methods

### General

H460 (human non-small cell lung cancer cells), HT-1080 (human fibrosarcoma connective tissue epithelial cells) and C6 (N-nitrosomethylurea-induced rat glioma cells) were obtained from ATCC (Manassas, VA) and maintained according to the provider's protocols. Cell lines were routinely confirmed free of mycoplasma contamination using a MycoAlert® Mycoplasma Detection Kit (Lonza, Basel, Switzerland). Cell lines were validated *via* Labcorp cell line authentication services (Labcorp, Burlington, NC, USA).

### Radiochemistry

[^18^F]hGTS13 was synthesized as previously reported 13.

### Incucyte live cell imaging

Cells (2.5 × 10^4^ per well) were seeded in 48-well plates the day before treatment with ferroptosis inducing drugs. On the day of treatment, cells were incubated in complete media containing BODIPY™ 581/591 C11 dye (BODIPY C11, 1.5 μM) and erastin2 (0, 340, 250, 200, 150, and 100 nM) or RSL3 (0, 300, 150, and 75 nM), and monitored for cell confluence and BODIPY C11 oxidation (total integrated intensity; green object CU × μm^2^) over the course of 24 h using an Incucyte SX1 live-cell analysis instrument (Sartorius AG, Göttingen, GER).

### Flow cytometry

Cells (1.7 × 10^5^ per well) were seeded in 6-well plates the day before ferroptosis induction. On the day of treatment, cells were incubated in complete media containing BODIPY C11 dye (1.5 μM) and erastin2 (150 nM) or RSL3 (300 nM) for 12 h and 2.5 h, respectively. Following treatment, cell monolayers were washed with HBSS, detached using TrypLE Express, and centrifuged to collect cell pellets. Pellets were then washed with HBSS to remove excess dye, resuspended in FACS buffer containing DAPI nuclear stain (0.4 µg/mL), and analyzed using an EMD Millipore Guava EasyCyte HT flow cytometer (MilliporeSigma, Burlington, MA., USA). Flow cytometry analysis was performed using FlowJo v10.9.0.

### Glutathione quantification

Cells (1 × 10^4^ cells per well) were seeded in 96-well plates the day before ferroptosis induction. Cells were then treated with erastin2 (150 nM) or RSL3 (300 nM) for 12 h and 2.5 h, respectively, in complete media. Total (GSH + GSSG) and oxidized (GSSG) glutathione were then quantified using a GSH/GSSG-Glo^TM^ assay (Promega, Madison, WI, USA), and the reduced (GSH) glutathione contribution to the total glutathione pool was determined using these data.

### [^18^F]hGTS13 uptake studies

Cells (1 × 10^5^ per well) were seeded in twelve-well plates the day before uptake studies (n=4 per condition). On the day of the uptake, cells were treated with erastin2 (150 nM) or RSL3 (300 nM) for 12 h and 2.5 h, respectively, in complete media. Erastin2- and RSL3-containing media was then removed, and pre-warmed Hanks balanced salt solution (HBSS) containing ~0.2 MBq/mL of [^18^F]hGTS13 was added to individual wells (1 mL per well). Cells were incubated at 37 °C and 5% CO_2_ for 1 h. Radiotracer media was then removed, and cell monolayers washed twice with 1 mL of HBSS. 200 µL RIPA lysis buffer containing Halt protease inhibitor was then added to each well and allowed to incubate on ice for 5 min. 150 µL of each cell lysate was then collected from each well and placed in a gamma counter tube. Radioactivity of cell lysates was determined using a Wizard^2^ 2480 automatic gamma counter (PerkinElmer, Waltham, MA, USA). Lysates were then frozen and allowed to decay overnight prior to total protein content quantification using a Pierce BCA protein assay kit (Thermo Fisher Scientific, Waltham, MA., USA).

### Animal studies

All experiments involving animals were in accordance with protocols approved by the Institutional Animal Care and Use Committee at Stanford University and were performed based on the NIH Guide for the Care and Use of Laboratory Animals. Male and female rats (n=5) utilized for toxicity studies were administered non-radiolabeled hGTS13 to determine any potential chemical toxicity. We employed a dose allometrically scaled to rats (31.9 µg/kg) that equates to 100× the maximum anticipated human dose (0.052 µg/kg for a 60 kg human). An additional group of rats (n=5) were administered a vehicle control (10% v/v ethanol in saline). All studies were completed at a specialized CRO (Jubilant Biosys) under Good Laboratory Practice conditions. Animals were observed for 14-days post dosing with an interim necropsy completed on day 2. All mortalities, clinical signs, time of onset, duration, and reversibility of toxicity were recorded. Study endpoints included body weight, clinical signs, clinical chemistry, hematology, and histopathology. A vehicle control group was similarly analyzed. The C6 syngeneic glioma models were developed as previously described 14. In brief, Sprague Dawley rats (n=6) were orthotopically implanted with 5 × 10^5^ C6 glioma cells into the right entorhinal cortex and tumor growth monitored by contrast-enhanced T1-weighted MRI using Gadavist. Approximately 4-weeks after implantation, rats underwent 60-min dynamic PET imaging immediately after intravenous administration of ~20 MBq [^18^F]hGTS13. PET images were acquired using a Siemens D-PET (matrix size 256 × 256; attenuation corrected). The dynamic PET data was acquired in list mode and reconstructed into 4 × 15, 4 × 60, 11 × 300 s frames. At the completion of the study, representative rat brains were harvested for *ex vivo* autoradiography and H&E analysis. PET imaging data was co-registered to the MRI for subsequent image analysis. No partial volume correction was completed. *In vivo* blocking of the system xc- transporter was performed using imidazole ketone erastin (IKE) (Catalog# HY-114481, MedChem Express, Monmouth Junction, NJ). IKE was prepared as a 15 mg/ml solution in 10% DMSO, 50% PEG-400, and 40% saline. Solubilization of IKE was achieved by first dissolving 6 mg IKE powder in 40 µL DMSO, followed by addition of 200 µL PEG-400, and then 160 µL saline in a drop-wise manner. Water bath sonication was performed immediately after the addition of each volume of solvent and each drop of saline to yield a homogenous, floc-free solution. C6 glioma-bearing rats (n=4) were administered ~20 MBq [^18^F]hGTS13, and PET images were acquired using a Siemens Inveon multimodality PET/CT scanner (Siemens, Munich). PET data was acquired in list mode and reconstructed into 4 × 300 s frames. 48 h later, the same rats were administered IKE intravenously under anesthesia at a dose of 25 mg/kg. IKE was allowed to circulate for 30 min before repeating the same PET imaging protocol. PET image analysis was performed using Inveon Research Workplace software. Three-dimensional regions of interest (ROIs) were constructed for tumors in the pre-blocking PET/CT scans. These 3D ROIs were then imported to the post-blocking PET/CT scans of the same rats and oriented to match the same regions from the pre-blocking scans. 3D ROIs for healthy brain were drawn on the contralateral brain tissue.

### Gene expression analysis

For human glioma tissue samples, informed consent was obtained under IRB protocol 12625, and then patient tissue used to asses xCT levels through Stanford IRB protocol 34363. Human autopsy brain samples were obtained from the Research Autopsy Center at Stanford under IRB protocol 63818. Human glioma and autopsy brain tissue samples were homogenized using micro-pestles, microcentrifuge tubes, and QIAshredder tissue homogenization spin-columns (Catalog# 79656, Qiagen). Total RNA was extracted and isolated using a GeneJet RNA Purification Kit (Catalog# K0731, Thermo Fisher Scientific) according to the manufacturer's protocol. First-strand cDNA synthesis was performed using a High-Capacity cDNA Reverse Transcription Kit (Catalog# 4368814, Thermo Fisher Scientific). Quantitative PCR was performed using a CFX96 Real-Time PCR System (Bio-Rad) and PowerTrack SYBR^®^ Green qPCR Master Mix (Catalog# A46012, Thermo Fisher). The mRNA encoding the light chain of system xc-, xCT (gene name: *SLC7A11*), was amplified using the following primers: (forward) 5'- CAAATGCAGTGG-CAGTGACC and (reverse) 5'- AGACAGCAAACACACCACCG, and 18S rRNA was amplified using: (forward) 5'-GTAACCCGTTGAACCCCATT and (reverse) 5'-CCATCCAATCGGTA-GTAGCG. The xCT mRNA transcripts were normalized to 18S rRNA. The comparative Ct method (ΔΔCt) was used to compare glioma samples to healthy brain tissue, and relative fold changes were determined using the 2^-ΔΔCt^ method.

### Statistical analyses

Data were expressed as mean ± standard deviation. Statistical significance was determined using a two-tailed Student's t test, *p*-value <0.05 was determined significant. For analysis across multiple samples, one-way analysis of variance (ANOVA) was used, followed by multiple comparisons of means with Bonferroni correction. Correlation between individual variables was determined using simple linear regression and two-tailed Pearson correlation coefficient (r). All statistical analyses were performed using GraphPad Prism ver. 10.2.2.

## Results

### Erastin2 induces ferroptosis in HT-1080 and C6 cells, but not in H460 cells

**Figure 2:**
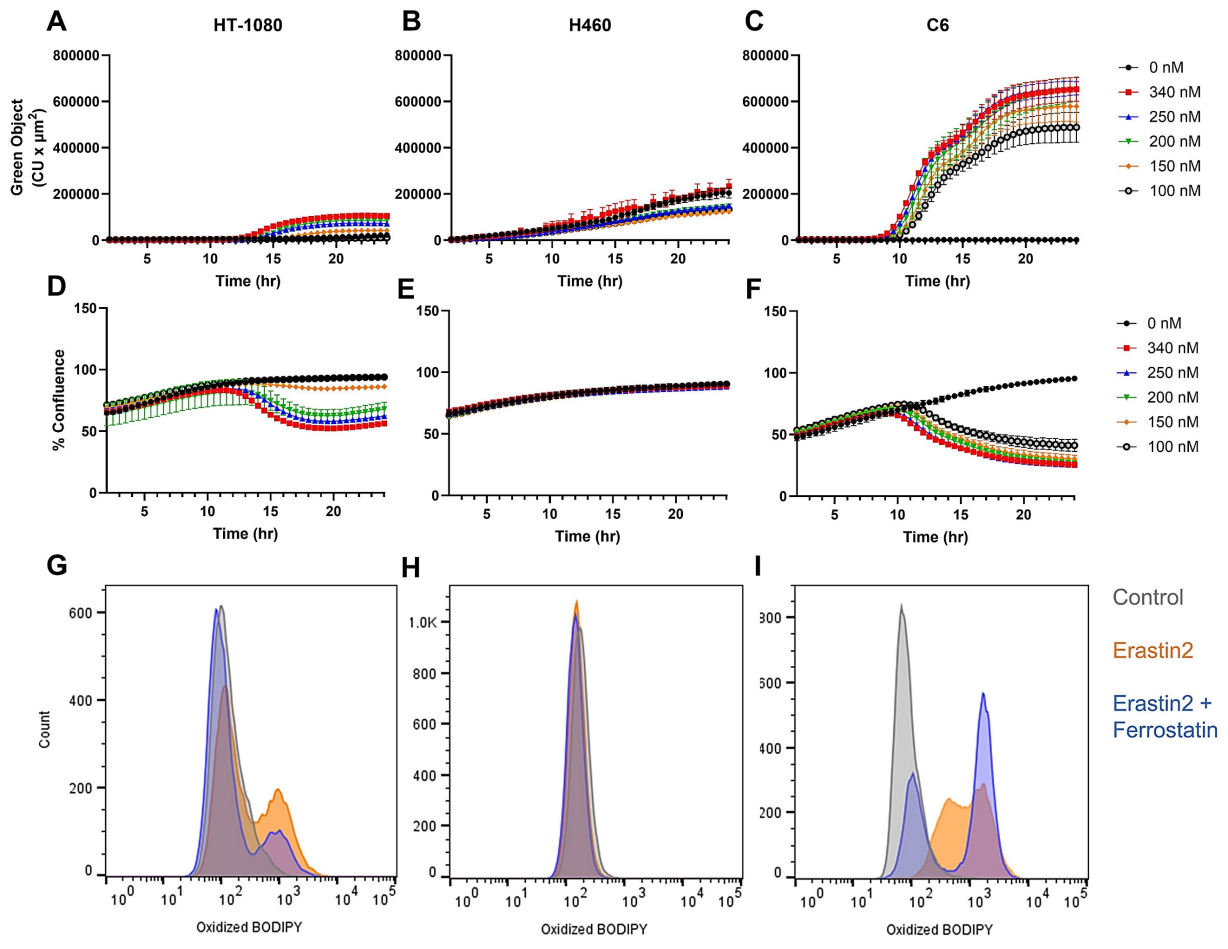
** Evaluation of erastin2-induced ferroptosis.** Overview of erastin2-induced ferroptosis using real-time live cell imaging and flow cytometry. **(A-C)** Plots of green object fluorescence in HT-1080, H460, and C6 cell lines, respectively, over 24h co-incubation with varying concentrations of erastin2 and 1.5 µM BODIPY 581/591 C11 lipid peroxidation sensor. **(D-F)** Corresponding plots of cell confluence resulting from treatments in A-C. **(G-I)** Flow cytometric analyses of HT-1080, H460, and C6 cell lines. Treatments: 0 μM erastin2 (grey); 150 nM erastin2 (orange); 150 nM erastin2 + 1 μM ferrostatin-1 (blue). All treatments were co-incubated with 1.5 µM BODIPY 581/591 C11. Data represent mean +/- SD. (n = 4, each condition).

We first set out to identify a set of cell lines where we could induce the production of pro-ferroptotic lipid peroxides while maintaining cell viability in order to adequately assess radiotracer uptake in live cells. Real-time live cell imaging using Incucyte revealed a dose-dependent increase in green object fluorescence and concurrent decrease in cell confluence when HT-1080, a ferroptosis-sensitive human fibrosarcoma cell line, was incubated with varying concentrations of erastin2, a potent ferroptosis inducer, and 1.5 μM BODIPY C11 dye in complete media (Figure 2A,D). BODIPY C11 is a fatty acid analog used as a lipid peroxidation sensor. The excitation and emission maxima of BODIPY C11 shifts from 581/591 nm (red) to 488/510 nm (green) upon oxidation by lipid peroxides. This apparent increase in accumulation of lipid peroxide was also observed in flow cytometric analysis of HT-1080 cells treated with 150 nM erastin2 for 12 h and subsequently stained with BODIPY C11, as evidenced by a shift in cell population along the x-axis representing oxidized BODIPY C11 fluorescence in the green channel (orange) (Figure 2G). Under these conditions, only 3% cell death was observed, while 35% of the viable cell population stained positive for lipid peroxide accumulation (Figure S1A- B). Coincubation of erastin2-treated HT-1080 with 1 μM ferrostatin-1, a lipophilic radical trapping antioxidant, partially suppressed ferroptosis in HT-1080 (blue) (Figure 2G). The addition of ferrostatin-1 decreased cell death to 2.6%, and cells staining positive for BODIPY C11 oxidation to 18% (Figure S1C). Conversely, H460 did not respond to erastin2 treatment when evaluated using either live cell imaging or flow cytometry. Although Incucyte green object fluorescence from oxidized BODIPY C11 increased over time in H460, the increase was virtually identical between control samples receiving no erastin2 and samples treated with the highest concentration tested (300 nM) (Figure 2B,E). Similarly, flow cytometric analysis of H460 revealed a negligible shift in the green channel following 12h treatment with 150 nM erastin2 (orange) (Figure 2H), with no perceived decrease in cell viability or increase in lipid peroxide accumulation, in comparison to untreated cells. (Figure S1D-E). C6, a rat glioma cell line, was the most sensitive of all cell lines to ferroptosis induction using erastin2. Live cell imaging of C6 revealed a dose-dependent response similar to that of HT-1080 following treatment with varying concentrations of erastin2, but with a much greater sensitivity to ferroptosis induction (Figure 2C). Real-time monitoring of cell confluence indicated that this accumulation of lipid peroxide also resulted in a more pronounced and prolonged negative effect on cell confluence than that observed in HT-1080 (Figure 2F). A similar trend was observed in flow cytometric analysis of oxidized BODIPY C11 in C6 cells, with a more dramatic shift of the cell population in the green channel following 12h treatment with 150 nM erastin2 (orange) (Figure 2I), and ~9% decrease in cell viability (Figure S1G-H). Coincubation with 1 μM ferrostatin-1 partially suppressed ferroptosis in erastin2-treated C6 cells (blue) (Figure 2I), slightly increasing cell viability, and decreasing the population of cells stained positive for oxidized BODIPY C11 (Figure S1I).

### RSL3 is a potent inducer of ferroptosis in sensitive cell lines

**Figure 3:**
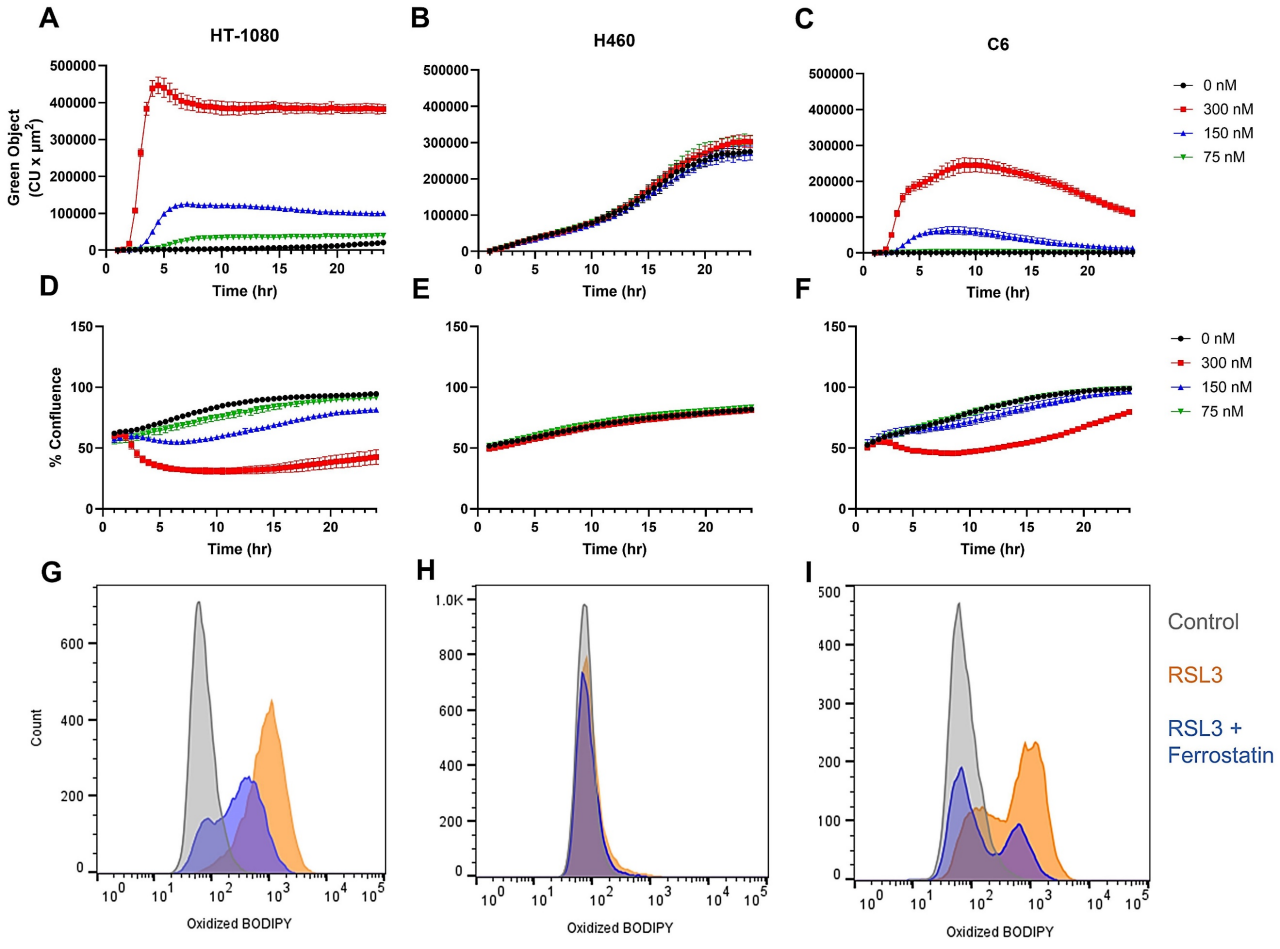
** Evaluation of RSL3-induced ferroptosis.** Overview of RLS3-induced ferroptosis using real-time live cell imaging and flow cytometry. **(A-C)** Plots of green object fluorescence in HT-1080, H460, and C6 cell lines, respectively, over 24h co-incubation with varying concentrations of RSL3 and 1.5 µM BODIPY 581/591 C11 lipid peroxidation sensor. **(D-F)** Corresponding plots of cell confluence resulting from treatments in A-C. **(G-I)** Flow cytometric analyses of HT-1080, H460, and C6 cell lines. Treatments: 0 μM RSL3 (grey); 300 nM RSL3 (orange); 300 nM RSL3 + 1 μM ferrostatin-1 (blue). All treatments co-incubated with 1.5 µM BODIPY 581/591 C11.

Having established the optimal doses and incubation times for the production of lipid peroxides while maintaining cell viability using erastin2, we next focused our attention on defining these parameters using the GPX4 inhibitor, RSL3, which exerts its inhibitory effects downstream of the system xc- transporter. Incubation of HT-1080 with varying concentrations of RSL3 resulted in a more rapid and pronounced increase in oxidized BODIPY C11 intensity than that observed during longitudinal treatment with erastin2 when evaluated using live cell imaging, and a similar, but inverse trend was observed with regard to cell confluence (Figure 3A,D). Flow cytometric analysis revealed an almost complete shift of cell population in the oxidized BODIPY C11 green channel following 2.5 h incubation with 300 nM RSL3 (orange), which was partially suppressed during coincubation with 1 μM ferrostatin-1 (blue) (Figure 3G). RSL3 treatment of HT-1080 resulted in ~91% of the cell population staining positive for oxidized BODIPY C11, while addition of ferrostatin-1 decreased this to ~52%. (Fig. S2B,C). H460, in agreement with its well-established resistance to ferroptosis, did not respond to treatment with RSL3. Although green object fluorescence increased over time in H460 treated with RSL3, there was no significant difference between control and treated samples at any concentration tested, and no effect on cell confluence was observed (Figure 3B,E). Flow cytometric analysis results mirrored that of live cell imaging, with a negligible shift of oxidized BODIPY C11 in the green channel (orange) that was not impacted by the addition of 1 μM ferrostatin-1 (blue) (Figure 3H). Treatment with RSL3 resulted in a negligible change in both cell viability and oxidized BODIPY C11 (Figure S2D-E). C6 responded to RSL3 treatment with an initial rapid increase in oxidized BODIPY C11 green object fluorescence, and eventual attenuation of this effect over time when incubated with 300 nM and 150 nM RSL3, as evidenced by live cell fluorescence imaging (Figure 3C). A decrease in cell confluence was observed at the higher concentration (300 nM), which was eventually recovered (Figure 3F). Flow cytometry plots of C6 treated with 300 nM RSL3 for 2.5 h revealed a clear shift of oxidized BODIPY C11 in the green channel (orange), with partial suppression of oxidized BODIPY C11 achieved during coincubation with 1 μM ferrostatin-1 (blue) (Figure 3I)**.** A decrease of ~7% cell viability was observed in C6 treated with RSL3 (Figure S2G-H).

### [^18^F]hGTS13 shows differential uptake in sensitive versus resistant cell lines

**Figure 4:**
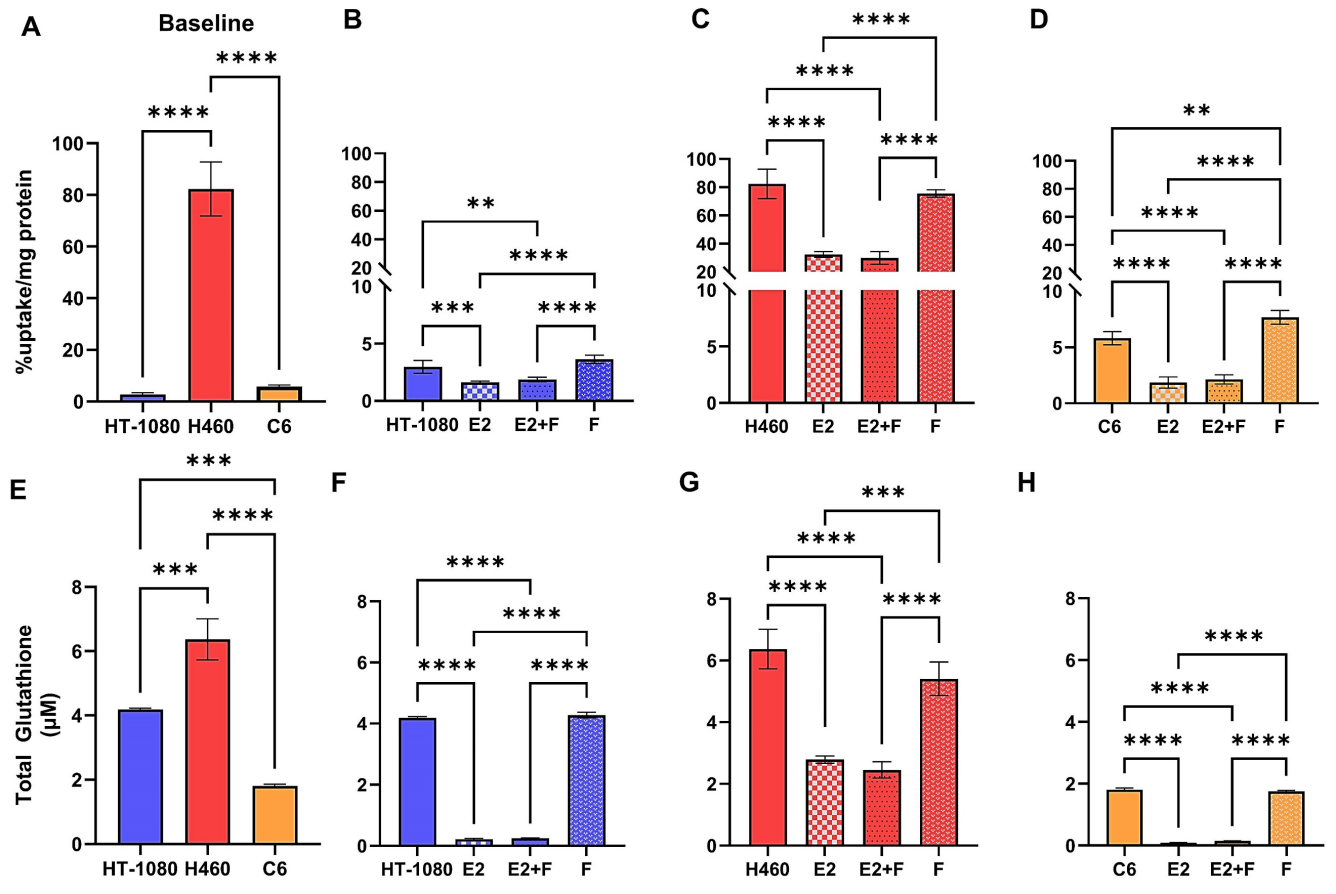
** [^18^F]hGTS13 uptake and glutathione production in the presence of erastin2.** Overview of differential radiotracer uptake and glutathione production in ferroptosis-sensitive and -resistant cell lines in response to treatment with erastin2. **(A)** Baseline radiotracer uptake in HT-1080, H460, and C6 cells. **(B-D)** Radiotracer uptake in HT-1080, H460, and C6 cells following treatment with erastin2 (E2) (150 nM) and/or ferrostatin-1 (F) (1 μM) for 12 h. **(E)** Baseline total glutathione (GSH+GSSG) content.** (F-H)** Total glutathione (GSH+GSSG) content of HT-1080, H460, and C6 following treatment with erastin2 (150 nM) and/or ferrostatin-1 (1 μM) for 12 h. **p < 0.01, ***p < 0.001, ****p < 0.0001, ns = not significant; 1-way ANOVA, multiple comparisons with Bonferroni correction. E2 = erastin2, F = ferrostatin-1.

We first sought to characterize the difference in baseline [^18^F]hGTS13 uptake across cell lines displaying sensitivity (HT-1080 and C6) or resistance (H460) to ferroptosis-inducing therapies by measuring cellular uptake over a period of 60-min (Figure 4A). We observed significant variation in radiotracer uptake across all cell lines (*p* < 0.0001). In ferroptosis-sensitive cell lines, HT-1080 and C6, we measured relatively low uptake of [^18^F]hGTS13 that was comparable across both cell lines. The respective uptake values in HT-1080 and C6 cells were 3.0 ± 0.6 %uptake/mg protein and 5.8 ± 0.6 %uptake/mg protein (n.s.). In contrast, we measured increased uptake in the H460 ferroptosis-resistant cells with values of 82.3 ± 0.6 %uptake/mg protein that was significantly higher than both HT-1080 (*p* < 0.0001) and C6 (*p* < 0.0001) cellular uptake.

### [^18^F]hGTS13 uptake is modified in the presence of erastin2

We next evaluated [^18^F]hGTS13 uptake in ferroptosis-sensitive (HT-1080 and C6) and ferroptosis-resistant (H460) cell lines in the presence of erastin2 (150 nM) and/or ferrostatin-1 (1 μM), and compared this to baseline uptake in the untreated cell lines (Figure 4). Erastin2 treatment of HT-1080 significantly decreased [^18^F]hGTS13 uptake by an average of 46% compared to baseline HT-1080 uptake (1.6 ± 0.1 vs. 3.0 ± 0.6 %uptake/mg protein, *p* = 0.001). Coincubation with ferrostatin-1 did not significantly rescue uptake in comparison to treatment with erastin2 alone (1.9 ± 0.2 vs. 1.6 ± 0.1 %uptake/mg protein, ns), and treatment with ferrostatin-1 alone resulted in [^18^F]hGTS13 uptake comparable to that of untreated HT-1080 cells (3.6 ± 0.4 vs. 3.0 ± 0.6 %uptake/mg protein, ns). H460 cells treated with erastin2 similarly displayed a 60.8% decrease in [^18^F]hGTS13 uptake compared to untreated cells (32.3 ± 2.1 vs. 82.3 ± 0.6 %uptake/mg protein, *p* < 0.0001). Intriguingly, treatment with erastin, the prototype ferroptosis inducer and less potent analog of erastin2, at a concentration of 5 μM for 5h, exerted minimal impact on [^18^F]hGTS13 uptake compared to untreated samples in HT-1080 (5.0 ± 0.4 vs. 4.9 ± 1.5 %uptake/mg protein, ns) or H460 (77.5 ± 4.0 vs. 88.7 ± 11.2 %uptake/mg protein, ns), but was effective at significantly reducing uptake in C6 (1.5 ± 0.03 vs. 3.2 ± 0.2 %uptake/mg protein, *p* < 0.0001) (Figure S3). Erastin also appeared to have little effect on HT-1080 and H460 in terms of lipid peroxide accumulation, even at a dose of 10 µM, but exerted a dramatic effect on C6 (Figure S4). No significant change in [^18^F]hGTS13 uptake was observed in H460 cells when comparing erastin2 with erastin2 + ferrostatin-1 treatment (32.3 ± 2.1 vs. 29.9 ± 4.4 %uptake/mg protein, ns), and treatment of H460 with ferrostatin-1 alone did not significantly impact tracer uptake compared to untreated cells (75.6 ± 2.6 vs. 82.3 ± 0.6 %uptake/mg protein, ns). Erastin2 had the most pronounced effect on [^18^F]hGTS13 uptake in C6 cells, decreasing uptake by 69.0% relative to untreated C6 cells (1.8 ± 0.5 vs. 5.8 ± 0.6 %uptake/mg protein, *p* < 0.0001) C6 cells treated with erastin2 + ferrostatin-1 resulted in a modest increase of tracer uptake, however this was not statistically significant in comparison to treatment with erastin2 alone (2.1 ± 0.4 vs. 1.8 ± 0.5 %, ns). Increasing the dose of ferrostatin-1 in the combinatorial treatment to 10 µM in a separate experiment significantly increased tracer uptake in C6 (3.8 ± 0.3 %uptake/mg protein) compared to untreated cells (3.2 ± 0.2 %uptake/mg protein, *p* = 0.0259) and cells treated with erastin2 (2.3 ± 0.3 %uptake/mg protein, *p* < 0.0001), but had no significant effect on either HT-1080 or H460 (Figure S5). Treatment of C6 with ferrostatin-1 alone significantly increased [^18^F]hGTS13 uptake by 32.8% to 7.7 ± 0.6 %uptake/mg protein (*p* = 0.0018), compared to untreated C6 (Figure 4D).

### Ferroptosis-sensitive and -resistant cell lines display disparate glutathione production, and inhibition of the system xc- transporter with erastin2 diminishes the glutathione pool

In order to place [^18^F]hGTS13 uptake values in the context of GSH production, we next assessed baseline total glutathione (GSH+GSSG) content of ferroptosis-sensitive and resistant cell lines (Figure 4E). We observed significant variation in total glutathione content across all cell lines (*p* < 0.01, *p* < 0.001). Total glutathione content of H460 (6.4 ± 0.6 μM) was significantly greater than that of both HT-1080 (4.2 ± 0.04 μM, *p* = 0.0003) and C6 (1.8 ± 0.05 μM, *p* < 0.0001) (HT-1080 vs C6: *p* = 0.0001).

We then assessed total glutathione (GSH+GSSG) content of ferroptosis-sensitive and ferroptosis-resistant cell lines following treatment with erastin2 and/or ferrostatin-1 (Figure 4F-H). Total GSH content of untreated HT-1080 cells (4.2 ± 0.04 μM) was significantly greater than that of HT-1080 treated with erastin2 (0.2 ± 0.03 μM, *p* < 0.0001) and erastin2 + ferrostatin-1 (0.2 ± 0.01 μM, *p* < 0.0001). Treatment with ferrostatin-1 only (4.3 ± 0.09 μM) had no significant impact on total GSH content in HT-1080 cells compared to untreated cells and, similar to the control samples, resulted in values significantly greater than that observed in cells treated with erastin2 and erastin2 + ferrostatin-1 (*p* < 0.0001). H460 followed a pattern similar to that of HT-1080 in each treatment group. Total GSH content of untreated H460 cells (6.4 ± 0.6 μM) was significantly higher than that of H460 treated with erastin2 (2.8 ± 0.1 μM, *p* < 0.0001) and erastin2 + ferrostatin-1 (2.5 ± 0.3 µM, *p* <0.0001). Treatment with ferrostatin-1 only (5.4 ± 0.5 μM) had no significant impact on total GSH content compared to untreated cells and, similar to the control samples, resulted in values significantly greater than that observed in cells treated with erastin2 and erastin2 + ferrostatin-1 (*p* < 0.0001). Total GSH content of C6 cells was significantly higher in untreated cells (1.8 ± 0.05 µM) compared to cells treated with erastin2 (0.09 ± 0.01 µM, *p* < 0.0001) and cells treated with erastin2 + ferrostatin-1 (0.15 ± 0.006 µM, *p* < 0.0001). Treatment with ferrostatin-1 only (1.8 ± 0.03 μM) had no significant impact on total glutathione content compared to untreated cells and, similar to the control samples, resulted in values significantly greater than that observed in cells treated with erastin2 and erastin2 + ferrostatin-1 (*p* < 0.0001). Overall, changes in total GSH content in cells mirrored changes observed in [^18^F]hGTS13 uptake under the conditions studied, and correlation plots confirmed the association between these variables in HT-1080 (r = 0.91), H460 (r = 0.97), C6 (r = 0.97) (Figure S6).

### RSL3-treatment shows minimal impact on [^18^F]hGTS13 uptake

**Figure 5:**
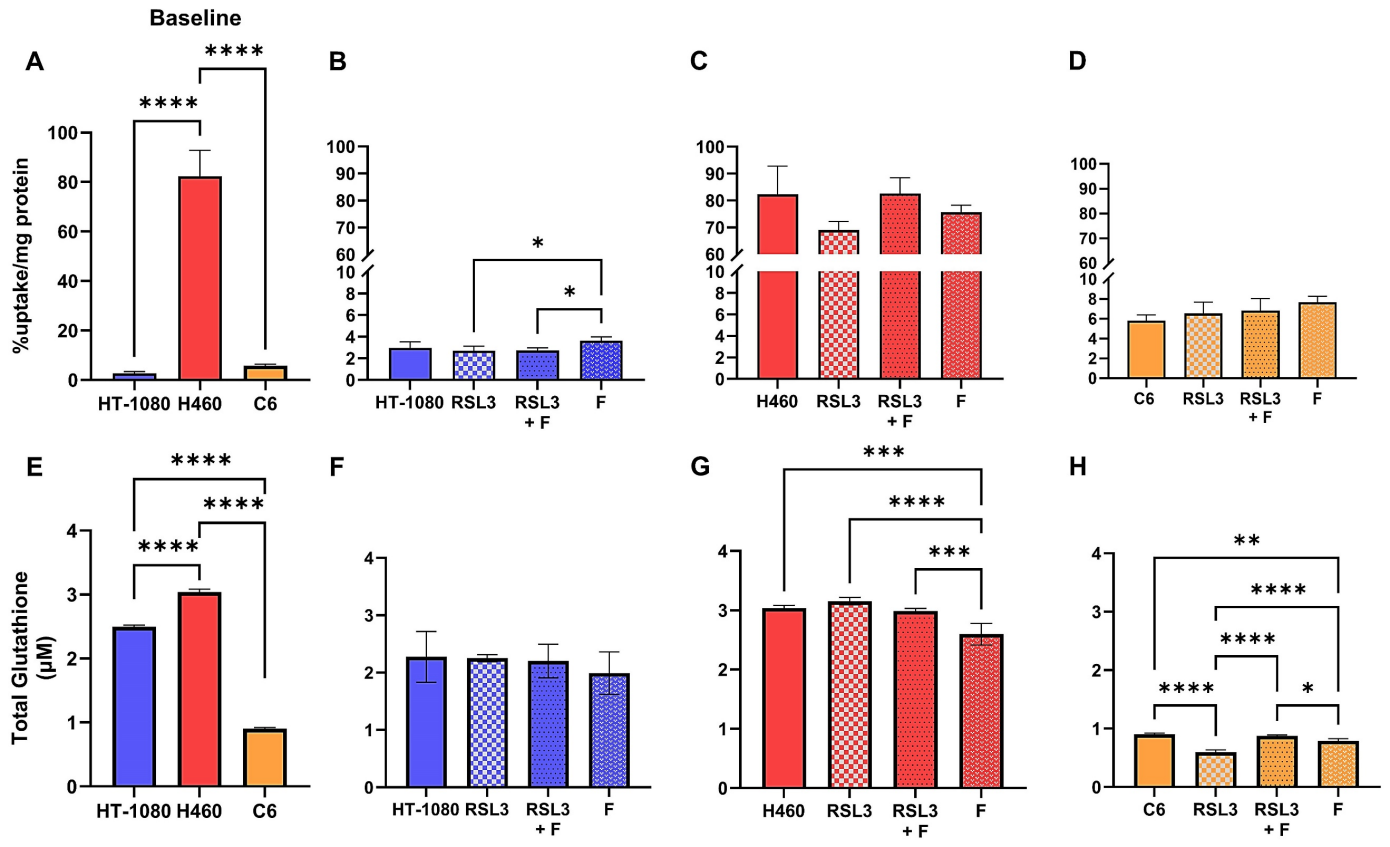
** [^18^F]hGTS13 uptake and glutathione production in the presence of RSL3.** Overview of differential radiotracer uptake and glutathione production in ferroptosis-sensitive and -resistant cell lines in response to treatment with RSL3. **(A)** Baseline radiotracer uptake in HT-1080, H460, and C6 cells. **(B-D)** Radiotracer uptake in HT-1080, H460, and C6 cells following treatment with RSL3 (300 nM) and/or ferrostatin-1 (1 μM) for 2.5h. **(E)** Baseline total glutathione (GSH+GSSG) content.** (F-H)** Total glutathione (GSH+GSSG) content of HT-1080, H460, and C6 following treatment with RSL3 (300 nM) and/or ferrostatin-1 (1 μM) for 2.5h. *p < 0.05, **p < 0.01, ***p < 0.001, ****p < 0.0001. ns = not significant. 1-way ANOVA, multiple comparisons with Bonferroni correction. F = ferrostatin-1.

We next evaluated the effect of the pro-ferroptotic GPX4 inhibitor RSL3 on [^18^F]hGTS13 uptake in the same cell lines compared to baseline uptake in untreated cells (Figure 5A-D). Treatment with 300 nM RSL3 for 2.5 h had no significant impact on tracer uptake relative to baseline values in all cell lines tested, regardless of their relative sensitivity/resistance to pro-ferroptotic compounds. The only significant change in tracer uptake observed was the increase in HT-1080 when treated with 1 μM ferrostatin-1 alone (3.6 ± 0.4 %uptake/mg protein), in comparison to treatment with RSL3 (2.7 ± 0.4 %uptake/mg protein, *p* = 0.0309) and RSL3 + ferrostatin-1 (2.7 ± 0.2 %uptake/mg protein, *p* = 0.0395) (Figure 5B).

### Inhibition of GPX4 with RSL3 impairs glutathione production only in C6

We then assessed the effects of RSL3 treatment on glutathione production across the same cell lines (Figure 5E-H). No significant difference in total glutathione content was observed in HT-1080 across treatments (Figure 5F). Interestingly, while RSL3 treatment had no significant effect on glutathione content in H460 compared to controls, treatment with 1 μM ferrostatin-1 alone (2.6 ± 0.2 µM) decreased total glutathione content significantly in comparison to untreated cells (3.0 ± 0.05 µM, *p* = 0.0003), RSL3 treatment alone (3.2 ± 0.06 µM, *p* < 0.0001), and RSL3 + ferrostatin-1 treatment (3.0 ± 0.04 µM, *p* = 0.0009) (Figure 5G). Only C6 responded significantly to treatment with RSL3 in terms of total glutathione content (0.6 ± 0.04 µM) in comparison to untreated cells (0.9 ± 0.02 µM, *p* < 0.0001), RSL3 + ferrostatin-1 treatment (0.9 ± 0.02 µM, *p* < 0.0001), and ferrostatin-1 treatment alone (0.8 ± 0.04 µM, *p* < 0.0001) (Figure 5H). Similar to the differences observed in H460, treatment of C6 with ferrostatin-1 alone resulted in a significant decrease in glutathione content compared to untreated cells (p = 0.0016) and cells treated with RSL3 + ferrostatin-1 (*p* = 0.0133) (Figure 5H).

### [^18^F]hGTS13 shows favorable imaging properties in rat models of glioma with no toxicity

**Figure 6:**
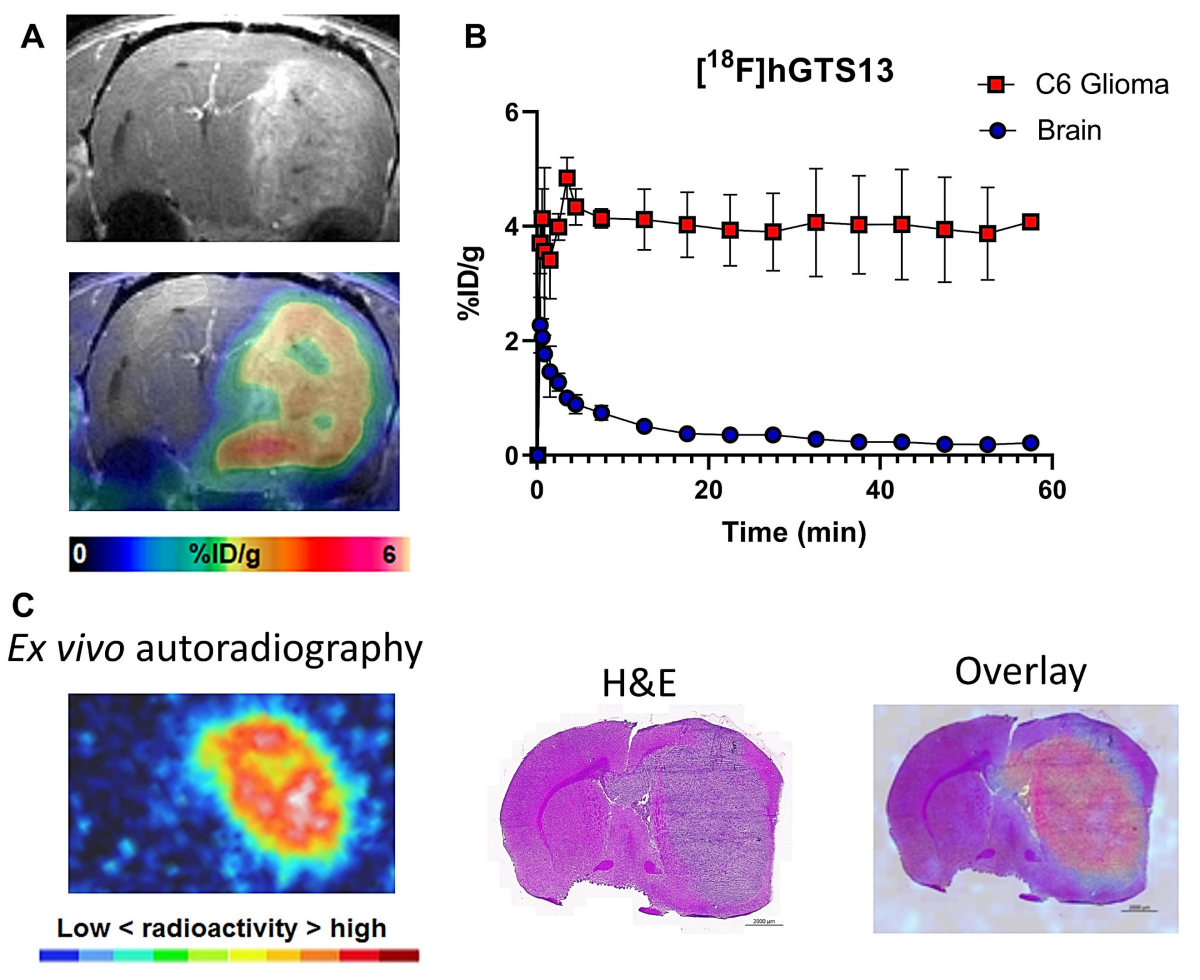
** [^18^F]hGTS13 Uptake in C6 orthotopic glioma-bearing rats.** *In vivo* and *ex vivo* evaluation of [^18^F]hGTS13 uptake and retention. **(A)** Representative [^18^F]hGTS13 MRI and fused PET/MRI image of orthotopic C6 glioma-bearing rat, summed 30 - 60 min post-injection. **(B)** Time activity curves of [^18^F]hGTS13 uptake in C6 glioma and healthy contralateral brain. **(C)** *Ex vivo* autoradiography, H&E staining, and corresponding overlay of an excised rat brain bearing an orthotopic C6 glioma following PET/MR imaging.

Non-radiolabeled hGTS13 was well-tolerated in cohorts of male and female rats (n = 5) at a dose allometrically scaled to 100× that of the maximum anticipated human dose, in comparison to a similarly analyzed vehicle control group (Table S2). No clinical signs of toxicity or changes in body weight were observed, and there were no consistent or correlative effects related to hGTS13 administration with regard to hematology, clinical chemistry parameters, coagulation parameters, urine analysis, and absolute or relative organ weights noted. No hGTS13-related histopathological findings were observed in this study. We next sought to validate the imaging potential of [^18^F]hGTS13 in a syngeneic, orthotopic C6 rat model of glioma. We completed dynamic PET/CT imaging of C6 glioma-bearing rats for 60-min beginning immediately prior to intravenous administration of 20 MBq of [^18^F]hGTS13. We observed that [^18^F]hGTS13 was characterized by high and sustained uptake within the intracranial gliomas (Figure 6A,B). Uptake within C6 gliomas peaked within the first few min post-injection of [^18^F]hGTS13 (4.8 ± 0.8%ID/g) and remained steady throughout the remainder of the scanning period (Figure 6B). In contrast, the contralateral normal brain tissue was characterized by initial tracer delivery which peaked within the first minute (2.3 ± 0.8%ID/g) post injection, and then rapidly cleared and remained low throughout the remaining duration of the scan (0.3 ± 0.07 and 0.2 ± 0.06%ID/g at the 30 and 60 min timepoints post injection, respectively). This resulted in a tumor-to-brain ratio (TBR) of 18.1 ± 4.8 at the 60 min timepoint (Figure 6B). *Ex vivo* autoradiography at the completion of the PET scan confirmed the imaging results and demonstrated elevated tracer uptake within the intracranial glioma in addition to minimal background signal in the surrounding brain tissue (Figure 6C). The pattern of radioactivity seen in the *ex vivo* autoradiography corresponded well with the presence of the intracranial glioma on the corresponding H&E section (Figure 6C).

### [^18^F]hGTS13 PET enables monitoring of pro-ferroptotic drug engagement *in vivo*

**Figure 7:**
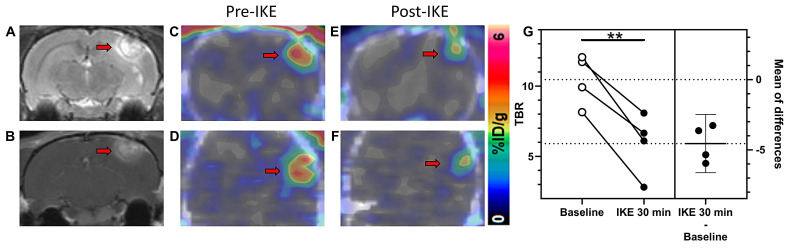
** [^18^F]hGTS13 PET-based monitoring of pro-ferroptotic drug engagement.** *In vivo* monitoring of drug engagement using [^18^F]hGTS13-PET. Representative **(A)** T1- and **(B)** T2-weighted MR images of C6 glioma-bearing rat. **(C)** Axial and **(D)** coronal PET/CT images of the same rat. **(E)** Axial and **(F)** coronal PET/CT images of the same rat 48 h later, following administration of IKE at 25 mg/kg and 30 min treatment. **(G)** Estimation plot of TBR before (baseline) and after IKE administration (n=4). **p < 0.01; Two-tailed Student's paired t test. Arrow = tumor.

Encouraged by the favorable tumor uptake of [^18^F]hGTS13 observed in glioma-bearing rats, we next evaluated the utility of the tracer to monitor the engagement of ferroptosis inducing drugs targeting system xc-. Given the fact that there was no substantial change in the time-activity curve (TAC) from 30-60 min following clearance of the tracer from the contralateral normal brain (Figure 6B), we subsequently employed a simplified imaging protocol involving 20 min static PET/CT scans of C6 glioma-bearing rats (n=4) during the 30-50 min timeframe post-injection of [^18^F]hGTS13. We first obtained baseline contrast-enhanced T1- and T2-weighted MRI (Figure 7A-B) and [^18^F]hGTS13 PET/CT (Figure 7C-D) imaging in a cohort of rats bearing C6 orthotopic gliomas (n=4). We then repeated imaging 48 h later following pre-treatment with a bolus of the system xc- inhibitor, imidazole ketone erastin (IKE, 25 mg/kg, administered 30-mins prior to intravenous administration of [^18^F]hGTS13) (Figure 7E-F). IKE was developed with increased metabolic stability and improved solubility relative to erastin2, making it the optimal choice for *in vivo* testing 15. [^18^F]hGTS13 PET imaging pre- and post-administration of IKE revealed a statistically significant decrease in mean TBR value from 10.5 ± 1.8 to 5.9 ± 2.2 (*p* = 0.006) (Figure 7G) indicating the potential for [^18^F]hGTS13 to monitor the engagement of ferroptosis-inducing drugs targeting system xc- *in vivo*.

### Disparate system xc- expression in human glioma samples implies varying sensitivity to ferroptosis-based therapies

**Figure 8:**
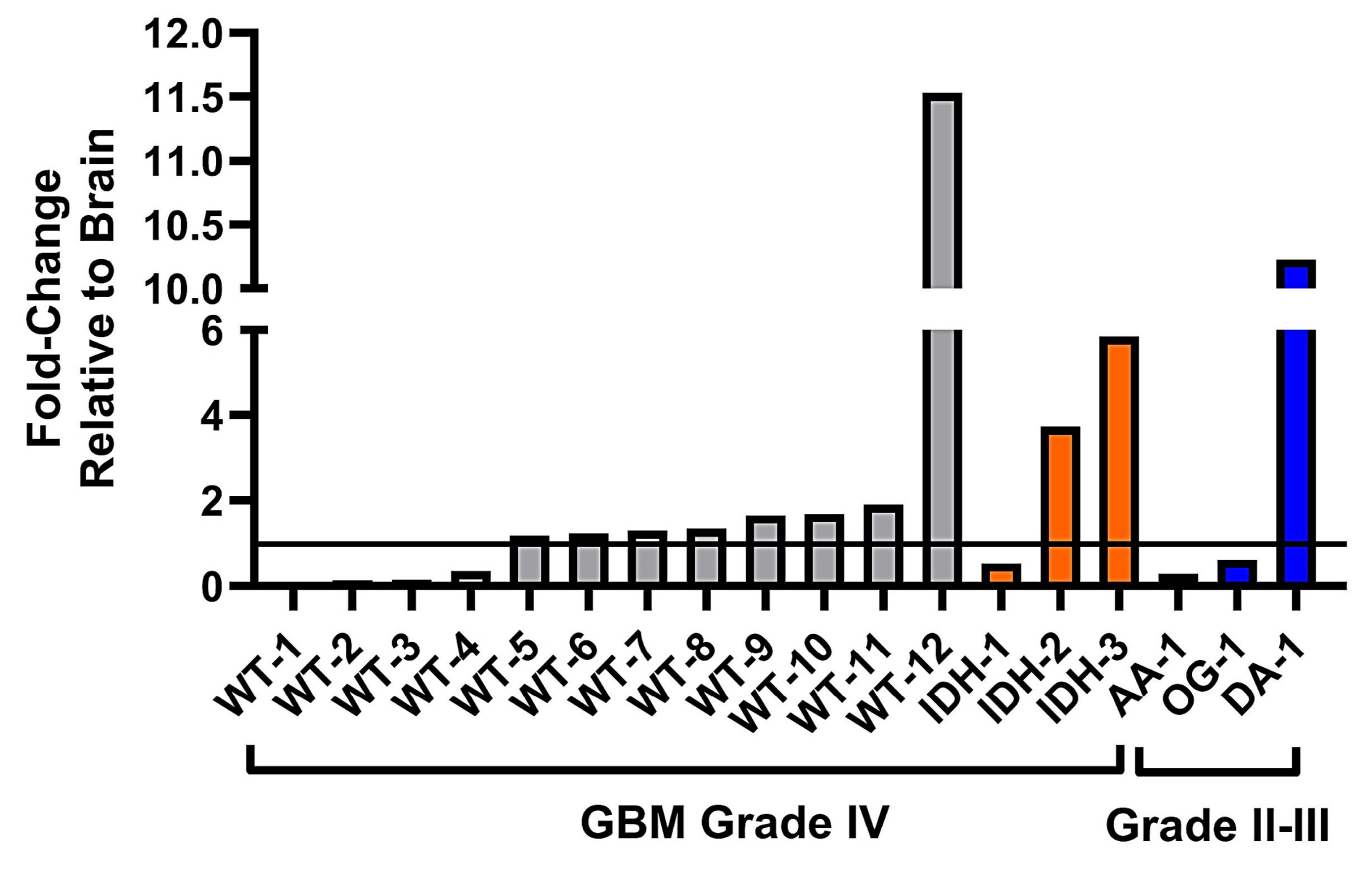
**System xc- expression in glioma subsets and healthy brain tissue.** Relative fold-changes in system xc- subunit *SLC7A11* mRNA expression of glioma subsets compared to healthy brain. WT = GBM, IDH-wild type; IDH = GBM, IDH-mutation; AA = anaplastic astrocytoma; OG = oligodendroglioma; DA = diffuse astrocytoma. Horizontal line bisecting graph = expression of xCT mRNA in healthy brain tissue; baseline expression set to a value of 1.

Lastly, we sought to investigate whether human glioma samples show a variation in system xc- expression and therefore would potentially display variation in sensitivity or resistance to ferroptosis-inducing drugs that specifically target the system xc- transporter. We completed the relative quantification of the light chain of system xc-, xCT, in surgical grade IV GBM samples varying in their isocitrate dehydrogenase (IDH) mutational status, compared to grade II-III glioma samples (Table S1), and brain tissue obtained from autopsy. Our analysis of GBM samples with IDH-wild type status revealed expression fold-changes of xCT ranging from 0.05× - 11.5× that of healthy brain (Figure 8). Notably, there were a group of samples (WT-1 - WT-4) with xCT expression below that of brain tissue, a further cluster of samples (WT-5 - WT-11) that demonstrated slightly elevated xCT expression, and lastly WT-12, that exhibited far greater xCT expression than all other samples. In the GBM samples with IDH-mutational status, there was similarly a range in xCT expression, from 0.5× - 5.9× that of autopsy brain, with IDH-2 and IDH-3 being characterized by moderate-to-high xCT expression. Lastly, the group of grade II-III glioma samples revealed similarly disparate expression of xCT, ranging from 0.3× - 10.2× that of healthy brain and sample DA-3 being defined as a high expressing sample.

## Discussion

Ferroptosis is a type of regulated cell death marked by the iron-dependent buildup of membrane lipid peroxides, leading to oxidative damage and the disruption of the plasma membrane 1. Targeting ferroptosis represents a novel approach to glioma therapy with promise to selectively activate this mechanism in cancer cells. Pharmacological induction of ferroptosis *via* inhibition of system xc- is an emerging class of anti-cancer therapy. However, there is a lack of predictive imaging biomarkers to define who would ultimately be suitable for ferroptosis-based therapy and monitor the engagement of pro-ferroptotic drugs *in vivo*.

The observed differences in radiotracer uptake between sensitive and resistant cell lines reported herein suggest that [^18^F]hGTS13 could be used to non-invasively distinguish tumors that would likely display sensitivity or resistance to pro-ferroptotic therapies, with resistant tumors being characterized by significantly higher baseline uptake of [^18^F]hGTS13. In addition, [^18^F]hGTS13 is effective at monitoring the engagement of a ferroptosis-inducing small molecule that exerts its inhibitory effects directly on the system xc- transporter, suggesting the utility of this strategy to monitor the engagement of such drugs as they advance to clinical investigation. Intriguingly, erastin2 was effective at inhibiting the system xc- transporter and [^18^F]hGTS13 uptake not only in sensitive cell lines, but also in the highly resistant H460 line. However, H460 cells in the presence of erastin2 still displayed ~30% uptake/mg protein; a value 10-fold greater than the baseline uptake observed in untreated HT-1080 and C6 cells. Furthermore, erastin, the less potent “prototype” analog of erastin2, was effective at reducing tracer uptake in C6, but exerted minimal impact on HT-1080 and H460, suggesting that inhibitory molecules of increasing potency will likely lead to greater transporter inhibition in cells that display considerable levels of tracer uptake, but minimal susceptibility to ferroptosis induction. The lack of tracer uptake rescue observed in cells treated with erastin2 + ferrostatin-1 is not surprising, as ferrostatin-1 exerts its anti-ferroptotic effects downstream of system xc-, and further reinforces the specificity of [^18^F]hGTS13 for the system xc- transporter. The decrease in total GSH content observed across all cell lines following treatment with erastin2 closely aligns with the differential uptake of [^18^F]hGTS13, suggesting that, in this context, the tracer uptake is reflective of the GSH pool. Consequently, this suggests that, in addition to monitoring drug engagement, [^18^F]hGTS13 could potentially be used to predict patient outcomes, assuming that erastin2 transporter engagement is indicative of the extent of ferroptosis induction. Because RSL3 operates independently of system xc-, [^18^F]hGTS13 was ineffective at monitoring RSL3-induced ferroptosis. There was no correlation between [^18^F]hGTS13 uptake and GSH content following treatment with RSL3. Therefore, the utility of this approach to identifying suitable patients for ferroptosis-based therapies and monitoring drug engagement depends on the identification of sensitive and resistant cell lines and monitoring the engagement of drugs that directly inhibit system xc-.

No clinical signs of toxicity or changes in body weight were observed in cohorts of male and female rats administered non-radiolabeled hGTS13 at a dose allometrically scaled to that of 100x the maximum anticipated human dose, in comparison to a cohort of rats administered vehicle control, furthering support for clinical translation of [^18^F]hGTS13. [^18^F]hGTS13 showed favorable and sustained tumor uptake in C6 glioma-bearing rats and minimal signal in the healthy contralateral brain, with a TBR of ~18 at 60 min post-injection. [^18^F]hGTS13 PET was also effective at monitoring IKE engagement with the system xc- transporter, as evidenced by the significant decrease in mean TBR values following *in vivo* inhibition of system xc- using IKE, albeit with some residual uptake remaining in the glioma lesions. Importantly, despite the statistically significant decrease in [^18^F]hGTS13 uptake following IKE treatment, it is worth noting that the IKE dose and treatment duration employed in this proof-of-concept *in vivo* study were not optimized for maximum effect and IKE is also not optimized for brain penetration. Future improvements in the solubility, potency, metabolic stability, and brain penetration of erastin analogs, and other system xc-inhibiting small molecules, will likely lead to greater inhibition of system xc- in living subjects, further advancing pro-ferroptotic therapies toward clinical use.

The variation in xCT expression observed across subsets of gliomas implies the occurrence of a wide range of GBM and grade II-III gliomas that would display varying sensitivity/resistance to pro-ferroptotic therapies that directly inhibit system xc-. As a system xc-specific imaging modality, [^18^F]hGTS13 PET has the potential to identify patients who would likely respond favorably to ferroptosis-inducing therapies that target system xc-, providing alternative treatment options for radio- and chemo-resistant gliomas. Based on the data described herein, we hypothesize that the samples characterized by elevated xCT expression (WT-12, IDH-2, IDH-3, and DA-1) may be resistant to ferroptosis-inducing therapies and be characterized by elevated glioma uptake of [^18^F]hGTS13. The question of whether this variation in [^18^F]hGTS13 radiotracer uptake holds true in a cohort of newly diagnosed GBM patients will be the focus of a future clinical investigation and careful consideration will be needed to define what parameters (standardized uptake values and/or TBR) constitutes lesions as having low/moderate or high uptake. Furthermore, the gene expression data does not take into account the metabolic demands occurring in cells and both of these aspects can influence radiotracer uptake.

One limitation of the current study is the use of a single rat glioma cell line. However, this study provides the proof of concept for employing [^18^F]hGTS13 in the context of glioma ferroptosis and we are currently investigating the sensitivity of other glioma cell lines, including those of patient derived origin. To our knowledge, this is the first report of molecular imaging in the context of ferroptosis-based cancer therapies, and these data establish a foundation for utilizing radiotracers specific to system xc- in order to differentiate ferroptosis-sensitive and -resistant cell lines and tumors, and to monitor the engagement of pro-ferroptotic drugs that directly target system xc-. Furthermore, this study represents a general framework for evaluating the use of such a molecular imaging approach in cancers beyond glioma.

## Conclusion

This study provides proof of concept data that [^18^F]hGTS13, a radiopharmaceutical specific for system xc-, can be employed to identify cell lines that will likely show sensitivity or resistance to ferroptosis inducing therapies targeting system xc-. While [^18^F]hGTS13 was effective at monitoring drug engagement of system xc- both *in vitro* and *in vivo*, there was no impact on radiotracer uptake upon treatment with ferroptosis inducing drugs with a mechanism of action independent of system xc- (e.g., RSL3). The variability of xCT expression in glioma samples suggests the potential for [^18^F]hGTS13 to identify subsets of patients who would likely derive benefit from ferroptosis-inducing therapies targeting system xc-. Clinical translation of [^18^F]hGTS13 is underway and defining the uptake parameters to classify patients with low or high tracer uptake will be the focus of future clinical investigation.

## Supplementary Material

Supplementary figures and tables.
