# Supplementary material for: Monitoring of cancer ferroptosis with [18F]hGTS13, a system xc- specific radiotracer
Source: Theranostics. 2025 Jan 1;15(3):836–49. doi: 10.7150/thno.101882 (PMC11700874; doi:10.7150/thno.101882)
Supplement: Supplementary file 1 — Supplementary figures and tables. [file thnov15p0836s1.pdf]

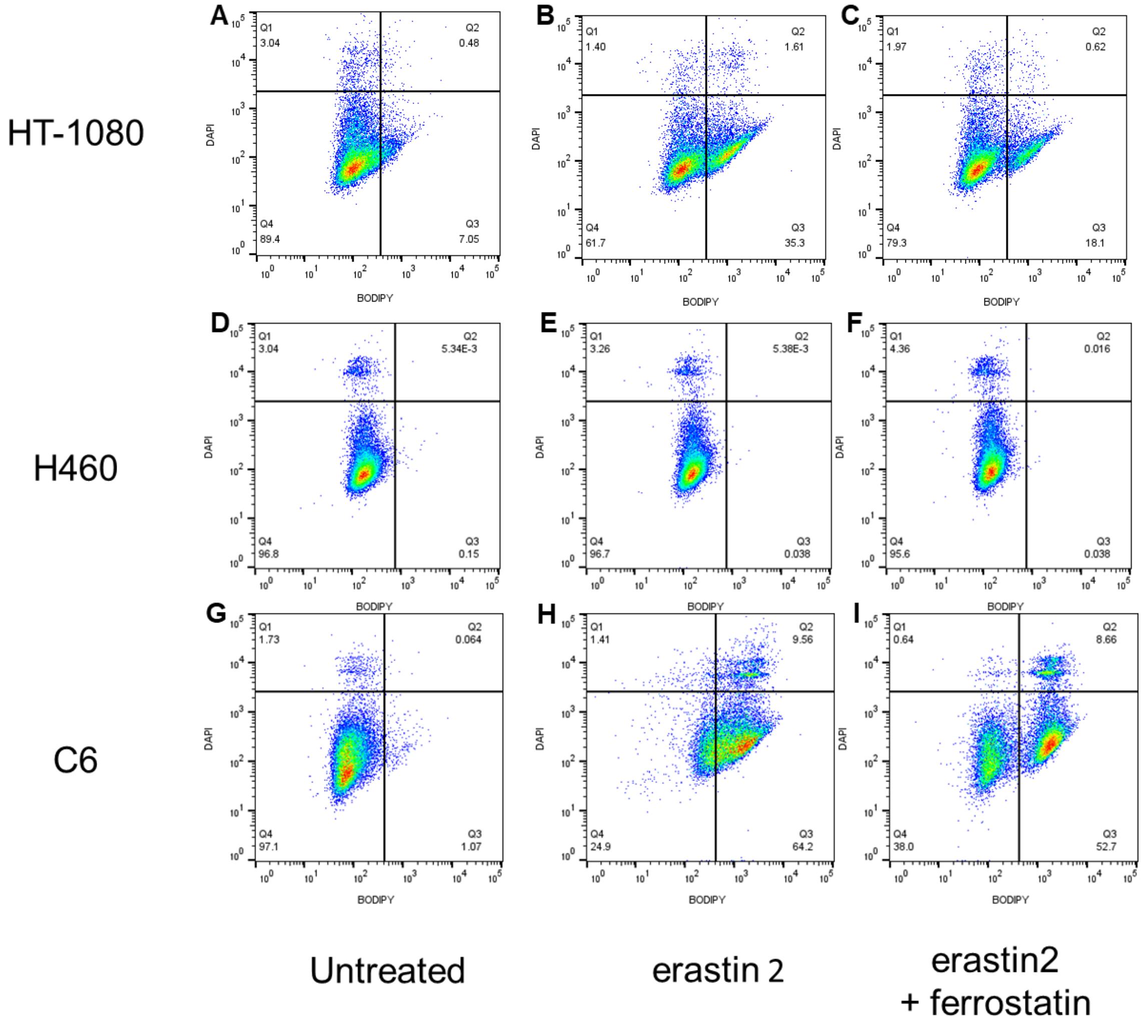

Figure S1. Flow cytometric analysis of erastin2 treatment.

(A-C) HT1080 cells incubated with complete media, erastin2 (0.15 µM), and erastin2 (0. 15µM) + ferrostatin (1 µM), respectively. **(D-F)** H460 cells incubated with complete media, erastin2 (0.15 µM), and erastin2 (0. 15µM) + ferrostatin (1 µM), respectively. **(G-I)** C6 cells incubated with complete media, erastin2 (0.15 µM), and erastin2 (0. 15µM) + f(1 µM), respectively. All samples treated for 5 h, co-incubated with BODIPY 581/591 C11 (1.5 mM), and stained with DAPI (0.4 mg/mL).

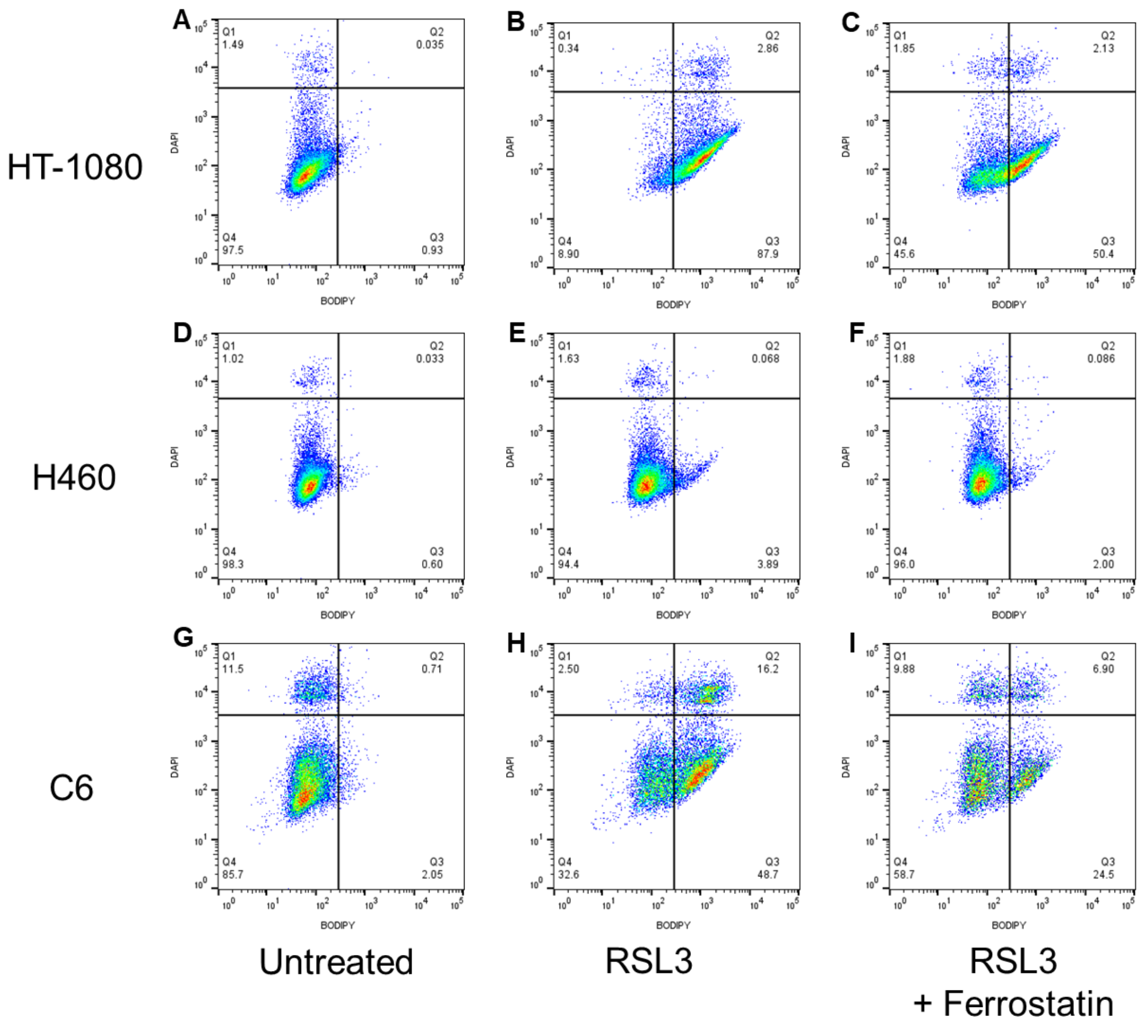

Figure S2. Flow cytometric analysis of RSL3 treatment.

(A-C) HT1080 cells incubated with complete media, RSL3 (0.3 μM), and RSL3 (0.3 μM) + ferrostatin (1 μM), respectively. **(D-F)** H460 cells incubated with complete media, RSL3 (0.3 μM), and RSL3 (0.3 μM) + ferrostatin (1 μM), respectively. **(G-I)** C6 cells incubated with complete media, RSL3 (0.3 μM), and RSL3 (0.3 μM) + ferrostatin (1 μM), respectively. All samples treated for 2.5 h, co-incubated with BODIPY 581/591 C11 (1.5 mM), and stained with DAPI (0.4 mg/mL).

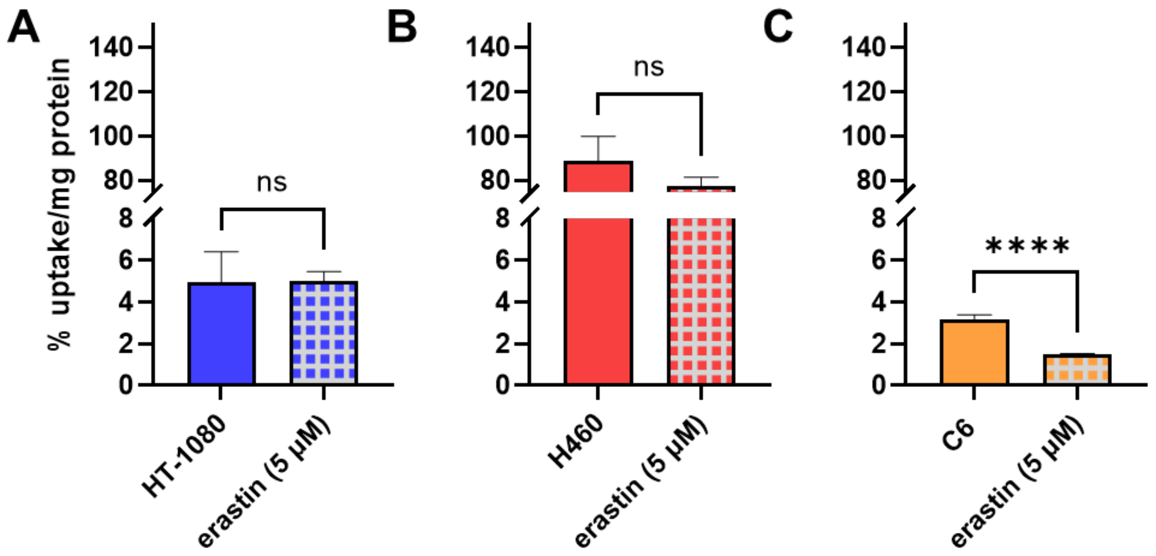

Figure S3. Radiotracer uptake in the presence of erastin.

[^{18}F]hGTS13 uptake of untreated **(A)** HT-1080, **(B)** H460, and **(C)** C6, and the same cell lines treated with Erastin (5 μM) for 5 h (n = 4). 1-way analysis of variance (ANOVA), multiple comparisons of means with Bonferroni correction.

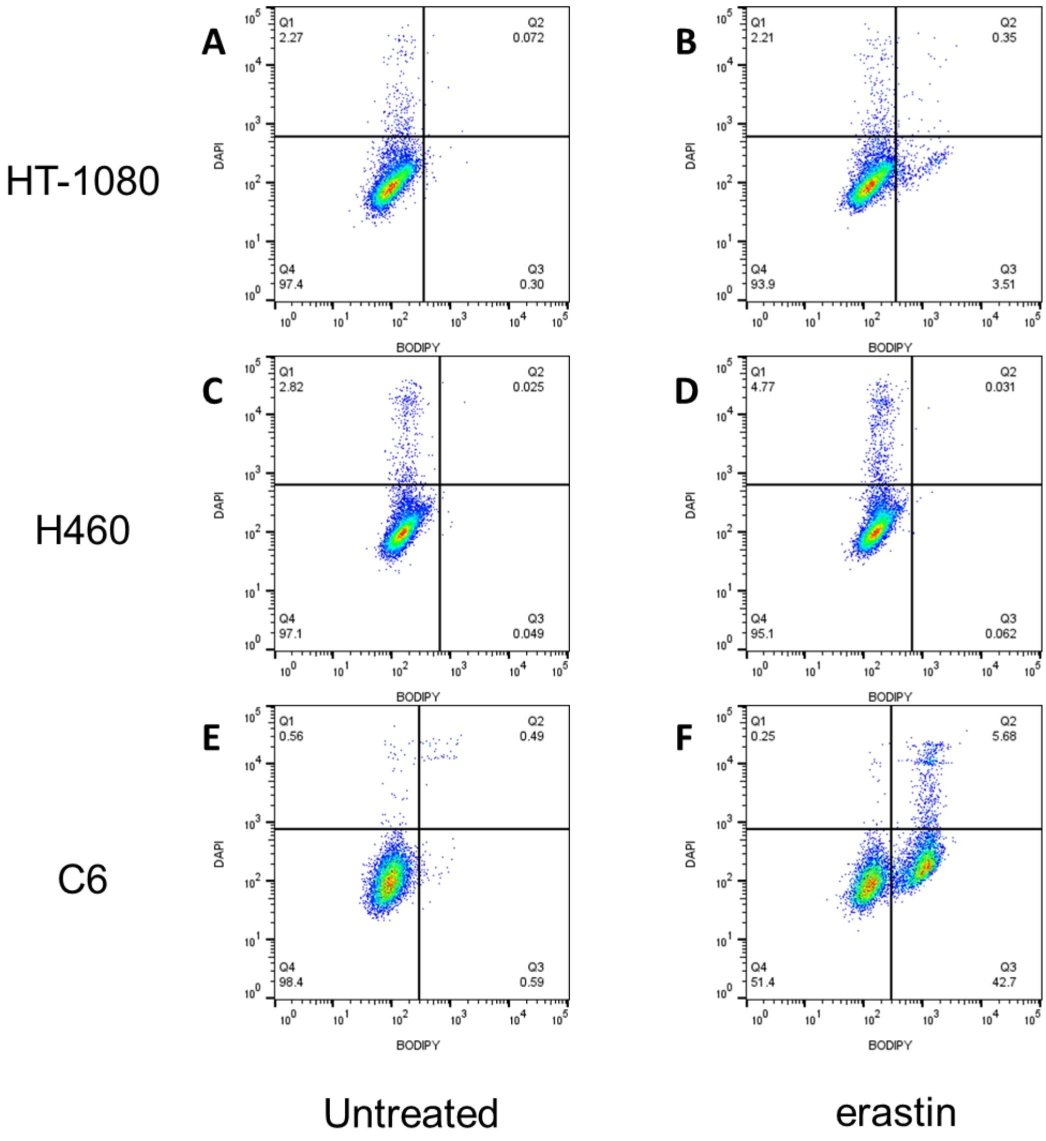

Figure S4. Flow cytometric analysis of erastin treatment.

(A,B) HT1080 cells incubated with complete media, and erastin (10 µM), respectively, for 5 h. **(C,D)** H460 cells incubated with complete media, and erastin (10 µM), respectively, for 5 h. **(E,F)** C6 cells incubated with complete media, and erastin (10 µM), respectively, for 5 h. All samples co-incubated with BODIPY 581/591 C11 (1.5 mM), and stained with DAPI (0.4 mg/mL).

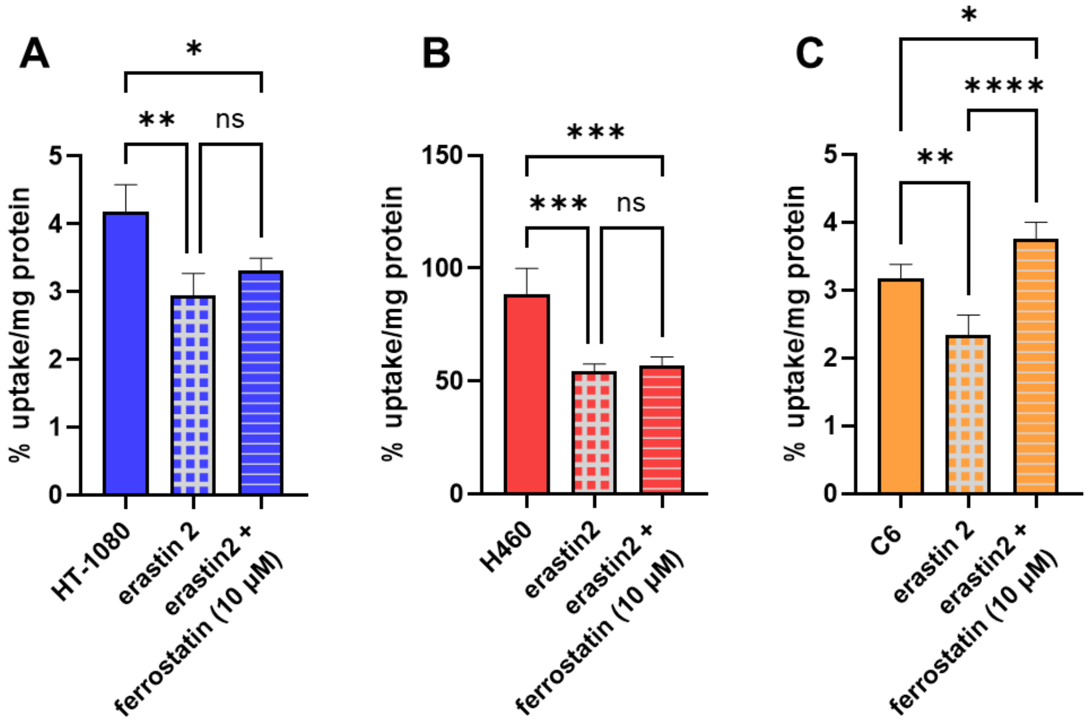

Figure S5. Radiotracer uptake in the presence of erastin2 and Increased ferrostatin.

[^{18}F]hGTS13 uptake of **(A)** HT-1080, **(B)** H460, and **(C)** C6 in complete media, erastin2 (0.15 µM), and erastin2 (0.15 µM) + ferrostatin (10 µM) (n = 4). 1-way analysis of variance (ANOVA), multiple comparisons of means with Bonferroni correction.

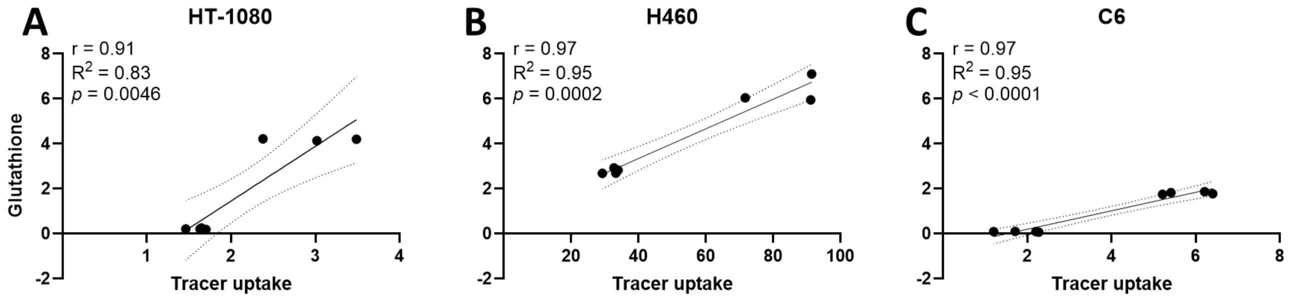

Figure S6. Correlation of radiotracer uptake and glutathione content.

Correlation plots of % radiotracer uptake / mg total protein vs. total intracellular glutathione concentration (µM) of **(A)** HT-1080, **(B)** H460, and **(C)** C6 cell lines in the presence and absence of erastin2 (0.15 µM). Simple linear regression using two-tailed Pearson correlation (r). Dotted lines represent 95% confidence interval.

Table S1. Patient demographic information

Sample ID	Age	Gender	Diagnosis	Primary/Recurrent sample
GBM WT-1	53	F	Glioblastoma, IDH1-wild type, Grade IV	Recurrent
GBM WT-2	62	F	Glioblastoma, IDH1-wild type, Grade IV	Recurrent
GBM WT-3	61	M	Glioblastoma, IDH1-wild type, Grade IV	Recurrent
GBM WT-4	61	M	Glioblastoma, IDH1-wild type, Grade IV	Primary
GBM WT-5	72	M	Glioblastoma, IDH1-wild type, Grade IV	Primary
GBM WT-6	69	M	Glioblastoma, IDH1-wild type, Grade IV	Primary
GBM WT-7	76	F	Glioblastoma, IDH1-wild type, Grade IV	Primary
GBM WT-8	79	M	Glioblastoma, IDH1-wild type, Grade IV	Recurrent
GBM WT-9	75	M	Glioblastoma, IDH1-wild type, Grade IV	Primary
GBM WT-10	63	M	Glioblastoma, IDH1-wild type, Grade IV	Primary
GBM WT-11	60	M	Glioblastoma, IDH1-wild type, Grade IV	Primary
GBM WT-12	69	M	Glioblastoma, IDH1-wild type, Grade IV	Recurrent
GBM WT-13	63	F	Glioblastoma, IDH1-wild type, Grade IV	Recurrent
GBM IDH-1	36	M	Glioblastoma, IDH1-mutant, Grade IV	Recurrent
GBM IDH-2	41	M	Glioblastoma, IDH1-mutant, Grade IV	Primary
GBM IDH-3	31	M	Glioblastoma, IDH1-mutant, Grade IV	Recurrent
AA-1	69	M	Anaplastic astrocytoma, IDH1, Grade III	Primary
OG-1	63	F	Anaplastic oligodendroglioma, IDH1-mutant, 1p/19q co-deleted, Grade III	Primary
DA-1	53	F	Diffuse astrocytoma, IDH1-mutant, Grade II	Primary
Brain tissue from autopsy	73	M	Cause of death: Acute fungal pneumonia contributing: Escherischia coli sepsis	

Table S2. hGTS13 Toxicity Data

Table 2.1 Hematology

	Day 3		Day 15	
	hGTS13: 31.9 µg/kg Males/Females	Vehicle Control: 0 µg/kg Males/Females	hGTS13: 31.9 µg/kg Males/Females	Vehicle Control: 0 µg/kg Males/Females
**Hematocrit**	38.0 ± 1.5/38.1 ± 2.0	40.0 ± 1.0/39.0 ± 1.9	41.9 ± 24/41.6 ± 1.9	43.0 ± 0.7/40.8 ± 1.1
**Hemoglobin**	13.4 ± 0.5/13.5 ± 0.7	14.0 ± 0.3/13.7 ± 0.6	14.8 ± 0.9/14.9 ± 0.6	15.2 ± 0.3/14.6 ± 0.3
**Mean corpuscular hemoglobin**	21.7 ± 0.4/21.3 ± 0.1	21.4 ± 0.6/20.6 ± 0.4	20.8 ± 0.5/21.1 ± 1.3	20.5 ± 0.3/20.6 ± 0.6
**Mean corpuscular hemoglobin concentration**	35.2 ± 0.2/35.4 ± 0.3	35.1 ± 0.1/35.1 ± 0.4	35.4 ± 0.4/25.9 ± 0.4	35.3 ± 0.3/35.9 ± 0.5
**Mean corpuscular volume**	61.8 ± 1.1/60.3 ± 0.4	61.2 ± 1.7/58.7± 0.9	58.8 ± 1.8/58.8 ± 3.1	58.3 ± 1.1/57.4 ± 1.1
**Platelets**	1109.0 ± 101.3/1032.6 ±185.6	974.6 ± 101.1/991.4 ± 389.6	1020.4 ± 52.3/888.8 ± 137.1	986.4 ± 38.8/1061.4 ± 64.6
**Mean Platelet volume**	6.0 ± 0.2/6.6 ± 0.4	6.3 ± 0.4/6.4 ± 0.4	6.0 ± 0.3/6.4 ± 0.2	5.9 ± 0.2/6.2 ± 0.2
**Red blood corpuscles**	6.16 ± 0.32/6.32 ± 0.31	6.55 ± 0.32/6.66 ± 0.42	7.13 ± 0.36/7.11 ± 0.67	7.37 ± 0.24/7.12 ± 0.29

**Red cell distribution width**	17.5 ± 1.7/17.1 ± 1.2	16.4 ± 0.5/16.7 ± 1.8	14.1 ± 0.7/13.3 ± 0.8	14.1 ± 0.8/13.9 ± 0.5
**White blood corpuscles**	5.35 ± 1.08/3.66 ± 0.34	5.83 ± 0.82/3.54 ± 0.75	8.27 ± 0.6/5.54 ± 1.10	6.99 ± 1.06/4.31 ± 1.29
**Neutrophils**	0.58 ± 0.24/0.29 ± 0.04	0.68 ± 0.24/0.54 ± 0.40	1.04 ± 0.13/0.63 ± 0.13	0.90 ± 0.16/0.51 ± 0.13
**Lymphocytes**	4.62 ± 0.91/3.27 ± 0.31	4.96 ± 0.81/2.85 ± 0.53	6.82 ± 0.61/4.68 ± 0.98	5.75 ± 1.24/3.63 ± 1.16
**Monocytes**	0.11 ± 0.05/0.06 ± 0.03	0.15 ± 0.03/0.10 ± 0.04	0.33 ± 0.06/0.16 ± 0.04	0.27 ± 0.06/0.13 ± 0.05
**Eosinophils**	0.03 ± 0.01/0.02 ± 0.02	0.04 ± 0.02/0.04 ± 0.02	0.07 ± 0.05/0.07 ± 0.02	0.07 ± 0.01/0.04 ± 0.01
**Basophils**	0.00 ± 0.00/0.00 ± 0.00	0.00 ± 0.00/0.00 ± 0.00	0.00 ± 0.00/0.00 ± 0.00	0.00 ± 0.00/0.00 ± 0.00
**Reticulocyte count**	4.12 ± 0.40/3.41 ± 0.50	4.37 ± 0.34/3.32 ± 0.44	3.59 ± 0.49/3.55 ± 0.43	3.62 ± 0.41/3.41 ± 0.33

Table 2.2 Clinical chemistry

	Day 3		Day 15	
	**hGTS13: 31.9 µg/kg Males/Females**	**Vehicle Control: 0 µg/kg Males/Females**	**hGTS13: 31.9 µg/kg Males/Females**	**Vehicle Control: 0 µg/kg Males/Females**
**Glucose**	161 ± 0.78/150.3 ± 12.2	161.3 ± 13.3/156.5 ± 7.6	111.4 ± 5.4/114.0 ± 5.4	111.3 ± 9.6/119.7 ± 7.5
**Alkaline phosphatase**	261.6 ± 44.3/170.4 ± 34.5	303.3 ± 43.2/157.7 ± 17.7	242.1 ± 32.9/153.8 ± 51.6	234.0 ± 40.2/140.5 ± 26.6
**Alanine aminotransferase**	43.5 ± 9.5/29.3 ± 4.6	42.4 ± 3.0/27.4 ± 2.0	42.4 ± 10.3/33.0 ± 4.9	42.7 ± 4.4/27.3 ± 5.0
**Aspartate aminotransferase**	73.2 ± 7.4/66.5 ± 4.2	73.1 ± 8.5/59.8 ± 5.1	76.2 ± 15.8/65.0 ± 6.5	81.9 ± 8.3/57.2 ± 11.3
**Gamma glutamyl transferase**	3.3 ± 1.9/4.1 ± 1.2	2.6 ± 1.6/6.1 ± 1.4	4.6 ± 2.7/6.1 ± 1.7	3.0 ± 1.6/7.2 ± 2.9
**Total protein**	4.72 ± 0.13/4.94 ± 0.24	4.84 ± 0.15/5.02 ± 0.13	5.3 ± 0.2/5.44 ± 0.3	5.37 ± 0.11/5.52 ± 0.26
**Albumin**	3.27 ± 0.06/3.50 ± 0.19	3.34 ± 0.15/3.46 ± 0.11	3.54 ± 0.09/3.68 ± 0.13	3.60 ± 0.07/3.68 ± 0.16
**Globulin**	1.49 ± 0.08/1.43 ± 0.12	1.50 ± 0.06/1.52 ± 0.07	1.76 ± 0.11/1.78 ± 0.17	1.75 ± 0.11/1.82 ± 0.1

**Albumin/Globulin ratio**	2.20 ± 0.11/2.45 ± 0.25	2.23 ± 0.06/2.27 ± 0.13	2.02 ± 0.07/2.08 ± 0.14	2.05 ± 0.15/2.02 ± 0.08
**Total bilirubin**	0.1 ± 0.02/0.08 ± 0.01	0.09 ± 0.03/0.01 ± 0.01	0.17 ± 0.02/0.19 ± 0.14	0.18 ± 0.04/0.19 ± 0.06
**Total cholesterol**	87.9 ± 4.3/89.6 ± 15.4	77.6 ± 5.0/102.9 ± 13.8	79.6 ± 10.4/103.6 ± 20.9	74.8 ± 6.4/96.3 ± 9.4
**Triglycerides**	41.8 ± 16.1/15.5 ± 7.2	28.9 ± 9.8/24.6 ± 6.6	44.5 ± 11.4/34.5 ± 2.8	37.7 ± 18.0/28.1 ± 6.4
**Urea**	39.2 ± 4.9/34.3 ± 5.1	38.3 ± 3.9/33.8 ± 8.7	33.5 ± 5.3/40.3 ± 5.9	33.8 ± 4.3/47.8 ± 5.8
**Creatinine**	0.43 ± 0.1/0.43 ± 0.08	0.43 ± 0.11/0.6 ± 0.16	0.43 ± 0.03/0.55 ± 0.14	0.39 ± 0.09/0.49 ± 0.05
**Creatine Kinase**	309.0 ± 39.4/217.9 ±27.4	332.4 ± 53.7/268.1 ± 29.0	258.1 ± 49.5/190.3 ± 29.7	285.0 ± 45.2/165.7 ± 32.1
**Sodium**	138.4 ± 0.9/137.5 ± 0.9	136.8 ± 1.3/137.5 ± 0.9	137.7 ± 1.3/138.0 ± 0.9	138.7 ± 1.0/137.2 ± 0.6
**Potassium**	4.57 ± 0.27/4.13 ± 0.2	4.59 ± 0.27/4.39 ± 0.26	4.40 ± 1.4/4.12 ± 0.12	4.37 ± 0.17/4.12 ± 0.17
**Calcium**	11.0 ± 0.4/11.0 ± 0.2	11.2 ± 0.2/11.0 ± 0.3	11.4 ± 0.2/11.5 ± 0.4	11.5 ± 0.1/11.6 ± 0.3
**Chloride**	103.0 ± 2.0/102.9 ± 0.9	102.0 ± 1.3/103.6 ± 1.3	104.3 ± 1.0/104.6 ± 0.9	104.1 ± 2.1/104.2 ± 1.4
**Phosphorous**	7.5 ± 0.3/6.8 ± 0.3	8.3 ± 0.4/7.1 ± 0.6	8.0 ± 0.4/6.6 ±0.3	7.5 ± 0.3/7.2 ± 0.6

Table 2.3 Coagulation analysis

	Day 3		Day 15	
	**hGTS13: 31.9 µg/kg Males/Females**	**Vehicle Control: 0 µg/kg Males/Females**	**hGTS13: 31.9 µg/kg Males/Females**	**Vehicle Control: 0 µg/kg Males/Females**
**Prothrombin time (sec)**	16.1 ± 0.6/15.8 ± 0.3	15.5 ± 1.0/15.4 ± 0.5	16.7± 1.5/16.0 ± 0.5	17.0 ± 0.6/16.1 ± 1.0
**Activated partial thromboplastin time (sec)**	14.4 ± 2.2/11.9 ± 1.7	12.9 ± 1.1/13.0 ± 0.7	13.1 ± 1.4/11.3 ± 0.2	14.5 ± 1.8/10.7 ± 1.7

Table 2.4 Urine analysis

	Day 3		Day 15
	**hGTS13: 31.9 µg/kg Males/Females**	**Vehicle Control: 0 µg/kg Males/Females**	**hGTS13: 31.9 µg/kg Males/Females**
**Blood (RBC/µL)**	Negative (5/5)/Negative (5/5)	Negative (5/5)/Negative (5/5)	Negative (5/5)/Negative (5/5)
**Bilirubin (mg/dL)**	Negative (5/5)/Negative (5/5)	Negative (5/5)/Negative (5/5)	Negative (5/5)/Negative (5/5)
**Urobilinogen (mg/dL)**	Normal (5/5)/Normal (5/5)	Normal (5/5)/Normal (5/5)	Normal (5/5)/Normal (5/5[illegible]
**Ketone (mg/dL)**	Negative (5/5)/Negative (5/5)	Negative (5/5)/Negative (5/5)	Negative (5/5)/Negative (5/5)
**Protein (mg/dL)**	Negative (5/5)/Negative (5/5)	Negative (1/5), 10 (3/5), 30 (1/5)/Negative (5/5)	10 (2/5), 30 (2/5), 100 (1/5)/Negative (3/5), 10 (1/5), 30 (1/5)
**Nitrite**	Negative (5/5), Negative (5/5)	Negative (5/5)/Negative (3.5), Positive (2/5)	Negative (5/5)/Negative (5/5)
**Glucose (mg/dL)**	Negative (5/5)/Negative (5/5)	Negative (5/5)/Negative (5/5)	Negative (5/5)/Negative (5/5)
**pH**	7.5 ± 0.5/6.9 ± 0.65	6.5 ± 0.35/6.6 ± 0.42	6.9 ± 0.65/6.6 ± 0.42
**Specific gravity**	≤1.005/≤1.005	≤1.005-1.020/≤1.005-1.010	1.010-1.020/1.005-1.020
**Leukocytes**	10 (5/5)/Negative (5/5)	Negative (3/5), 10 (2/5)/Negative (5/5)	Negative (2/5), 10 (2/5), 7[illegible] (1/5)/Negative (5/5)
**Volume**	7.68 ± 1.85/6.58 ± 2.06	8.06 ± 1.98/4.54 ± 2.02	8.10 ± 6.41/10.10 ± 3.94
**Color**	Light yellow (5/5)/Light yellow (5/5)	Light yellow (5/5)/Light yellow (5/5)	Light yellow (5/5)/Light yellow (5/5)
